# A Rana Macrodactyla-Inspired Compliant Positioner with a New Hybrid Lever–Double-Rocker Amplifier for Precision Nanoindentation Systems

**DOI:** 10.3390/mi17101161

**Published:** 2026-10-01

**Authors:** Minh Phung Dang, Ngoc Hau Huynh, Hieu Giang Le

**Affiliations:** Faculty of Mechanical Engineering, Ho Chi Minh City University of Technology and Engineering, Ho Chi Minh City 700000, Vietnam

**Keywords:** compliant mechanism, compliant XY positioner, pseudo-rigid-body method, dynamic analysis, Firefly algorithm

## Abstract

This paper presents a novel compliant XY positioner inspired by the Rana macrodactyla, incorporating a new hybrid displacement amplifier. The positioner was developed to achieve good performance characteristics, including an appropriate resonant frequency, a suitable displacement amplification ratio, effective linear motion, and a compact design enabled by its symmetric structure. The positioner was constructed based on a novel hybrid displacement amplifier that integrated a lever displacement amplifier with a double-rocker mechanism employing circular joints. It also incorporated a parallel guidance mechanism with compliant rectangular joints. In addition, an integration approach of the pseudo-rigid-body method and Lagrange principle, and finite element analysis (FEA) was employed to promptly analyze the static-dynamic attributes of the proposed mechanism. The difference between the theoretical and simulated outcomes showed a 2.2% error, which was minimal and reliable for speedily analyzing the suggested positioner’s output characteristics. Secondly, the developed positioner’s key design variables were optimized using the Firefly optimization algorithm based on analytical equations to improve the output characteristics of the platform. The optimized model of the platform was manufactured using the CNC wire-cut electric discharge machining technique. The experimental and simulated frequencies were 188.4 Hz and 204.6 Hz, respectively, resulting in an 8.6% discrepancy between the two approaches. Furthermore, the experimental validation verifies the suggested positioner’s potential use in nanoindentation systems and validates a linear input–output displacement response. The proposed mechanism demonstrates significant potential for precision positioning in micro-nano engineering systems.

## 1. Introduction

Because of its ability to exert light loads and measure depths at the nanoscale, the nanoindentation testing device has been developed to provide a reliable analysis of hardness, elastic modulus, and creep. This technological item also enables the observation and analysis of the mechanical characteristics of organic/inorganic, hard, and flexible materials [1]. Advances in in situ nanoindentation testers have generated great interest in the development of tiny mechanical systems with improved performance. It is possible to incorporate compact-sized mechanisms into nanoindentation testing positioning systems for real-time detection of the mechanical characteristics of the specimen. These mechanisms are designed to be lightweight and require minimal assembly [1,2]. Another interesting point is that the in situ nanoindentation checking technique is of great importance when it comes to investigating the characteristics of bio-specimens utilized in bio-materials, including femur, prosthetic bone, and tooth [3].

The positioner used in nanoindentation checking currently relies on traditional technology, including bearings, shaft and hole fit, bronze bushings, step motors, lead screws, ball nuts, straight and helical gears, as well as rigid joints. As a result, it is challenging to achieve high positioning accuracy and precise motion transmission because of the negative consequences of traditional mechanisms, including friction, lubrication, corrosion, and backlash. Thus, the indentation process requires an accurate positioner to determine a position before the specimen’s properties are evaluated. Recent advances in precision engineering have placed greater emphasis on intelligent feedback mechanisms, system flexibility, and system-level constraints, alongside structural and actuation designs, which may affect the implementation and operation of sophisticated machinery. These changes indicate that assessing high-performance positioning systems now encompasses not only displacement amplification, stiffness, and resonant frequency, but also alignment with new intelligent control and supervisory systems [4]. In this broader framework, compliant mechanisms [5,6,7] have been widely adopted. This is due to their inherent advantages, including no wear, backlash, friction, or lubrication, as well as their cost-effectiveness and miniaturization [8,9,10]. Standard in situ nanoindentation devices use two main positioners. To be more specific, a refined Z-positioner guides and controls the indenter, and a refined stage positions the material samples before nanoindentation testing to assess the bio-specimen’s mechanical characteristics [11].

Given the aforementioned problems, several scientists have made significant progress in developing compliant positioners for the in situ nanoindentation technique for real-time detection of the mechanical features of the specimen. Specifically, Huang et al. developed a compliant platform controlling one direction, in accordance with a flexure one-lever displacement magnifier that was incorporated with elliptic flexure joints for assessing the characteristics of a material sample. Similarly, Rabe et al. improved a compact mechanism controlling one direction that was integrated into an in situ nanoscratch mechanism for the purpose of evaluating characteristics of the specimen [12]. Huang et al. proposed a compliant platform controlling one direction with a semi-bridge magnifier that controls the indenter for an in situ nanoindentation instrument [13]. For the purpose of evaluating the mechanical properties of the specimen based on the nanoindentation testing instrument, Dang et al. [14] suggested a 01-dof flexure mechanism for directing the indenter. Huang et al. [15] proposed nanoindentation testing equipment with a platform with an operating space of 12 × 12 µm XY positioner and a 40 µm Z positioner.

Several compliant positioners with various effective configurations have been designed for precise locating systems [16,17]. In addition, a piezoelectric actuator (PEA) is commonly used in these applications because of its outstanding precision and quick response time. One drawback of PEAs is their limited travel [18], which restricts the ability of compliant stages to achieve large output displacements. As a result, several displacement magnifiers were designed to enhance the output displacement of established structures. These include a single lever displacement amplifier [18], a symmetric compliant four-lever displacement amplifier, a compliant L-shape lever displacement amplifier [19], a compliant bridge-lever-bridge displacement amplifier [20], a bridge-lever displacement amplifier [21], a compliant rhombus-lever displacement amplifier [22,23], a compliant two-lever displacement amplifier [24], and a compliant bridge-Scott–Russell displacement amplifier [25].

To achieve higher performance, a number of scientists have focused their efforts on constructing flexure platforms by combining series-parallel kinematic chains based on the PEAs of various applications. In particular, the resonance frequency of a compliant XY positioner was established according to the compliance matrix approach for vibration-aided polishing with silicon carbide ceramic material by Gu et al. [26]. Du et al. [27] applied the dynamic stiffness matrix method based on D’Alembert’s principle to model the quality characteristics of the compliant XY stage. The quality response of a compliant XY positioner integrated with a four-lever amplification structure for vibration-assisted CNC milling of 2083 material was modelled using the PRBM method by Dang et al. [28]. Ling et al. employed the PRBM method to examine the dynamic attributes of the developed positioner for an accurate locating system [22]. In addition, modelling the dynamic properties of a suggested 02-dof platform for an accurate locating system was accomplished by Wu et al. [29] through the application of the kinetostatics calculation method. Based on these effective analytical methods, an integrated analytical methodology of the PRBM and Lagrange principle was proposed for modelling the quality characteristics of the advanced platform in this study.

In recent years, a significant number of researchers have investigated the integration of displacement amplifiers into flexure positioners to improve the operating travel of precision positioning systems. In particular, Wu et al. [29] created a four-lever displacement amplification mechanism with right-circular joints incorporated into a 02-dof flexure positioner for an accurate locating system. Gan et al. [30] used a bridge displacement magnifier with compliant rectangular joints in an XY stage for accurate positioning. Zhu et al. [31] implemented a symmetric Scott–Russell displacement magnifier incorporated into an XY stage for a very accurate locating system. Ding et al. [32] employed a one-lever displacement amplifier incorporated into a compliant positioner with three degrees of freedom, suitable for micro-nano manipulation.

Bio-inspired engineering has garnered heightened interest since biological systems provide sophisticated structural and functional methodologies that can be utilized for the creation of improved mechanical and sensing technologies. Recent research shows that biological concepts can advance position sensing, artificial sensory systems, adaptive robotic frameworks, and other engineered devices by translating biological morphology, sensing, or movement techniques into practical engineering designs [33,34,35]. In the domain of compliant mechanisms, this methodology is especially appealing since both biological musculoskeletal systems and compliant constructions fundamentally depend on regulated deformation, distributed flexibility, and synchronized movement. By substituting traditional stiff joints with elastic deformation, compliant mechanisms can further diminish friction, backlash, wear, and assembly demands while preserving compact structural designs. These traits strongly motivate the use of biological shapes as conceptual frameworks for precision positioning systems. Against this background, although many 2-dof compliant mechanisms have been developed, effective designs for integrating a nanoindentation tester remain elusive. Meanwhile, the integration of a Rana macrodactyla-inspired positioner and a new hybrid lever–double-rocker amplifier for a nanoindentation system remains insufficiently explored. Therefore, this paper proposes a compliant XY positioner in which the hind-leg morphology of Rana macrodactyla provides the conceptual basis for the mobile-platform support topology, while a hybrid lever–double-rocker mechanism increases displacement amplification. In addition, this proposed positioner may facilitate the attainment of a compact design, a significant displacement magnification ratio, and a favourable linear correlation between the input–output displacement values at the input and output points in the X and Y directions. A hybrid optimization approach is proposed to increase the output response of the mechanism employed for the nanoindentation testing positioning system. This approach is achieved by integrating the PRBM [36] with the Lagrange technique, FEA, and the Firefly optimization algorithm [37]. The novel designs presented in this study are summarized as follows.

- A novel compliant XY positioner whose mobile-platform support structure is modelled after the articulated hind-limb anatomy of Rana macrodactyla.
- A new amplifier mechanism of a symmetrical single-lever and double-rocker structure.

This research aims to create an innovative compliant positioner inspired by the hind-leg structure of Rana macrodactyla. The mechanism integrates a central movable platform and a new amplifier mechanism, merging a lever structure with a symmetric double-rocker mechanism to provide linear motion in two directions and achieve high performance that is suitable for nanoindentation systems. First, analytical modelling of the output attribute of the improved structure was established using a hybrid analytical approach combining the PRBM and the Lagrange technique. Subsequently, the theoretical accuracy was assessed by FEA. Next, the Firefly optimization algorithm optimized the key variables of the proposed structure to enhance the mechanism’s output response. Eventually, an optimal design of the proposed positioner was produced using the wire electric discharge machining method to validate the optimal and finite element analysis results. Figure 2 illustrates the practical application of the designed platform for precisely positioning the medical material samples within the nanoindentation testing instrument.

## 2. Conceptual Structure for a Novel XY Flexure-Based Positioner

**Figure 1:**
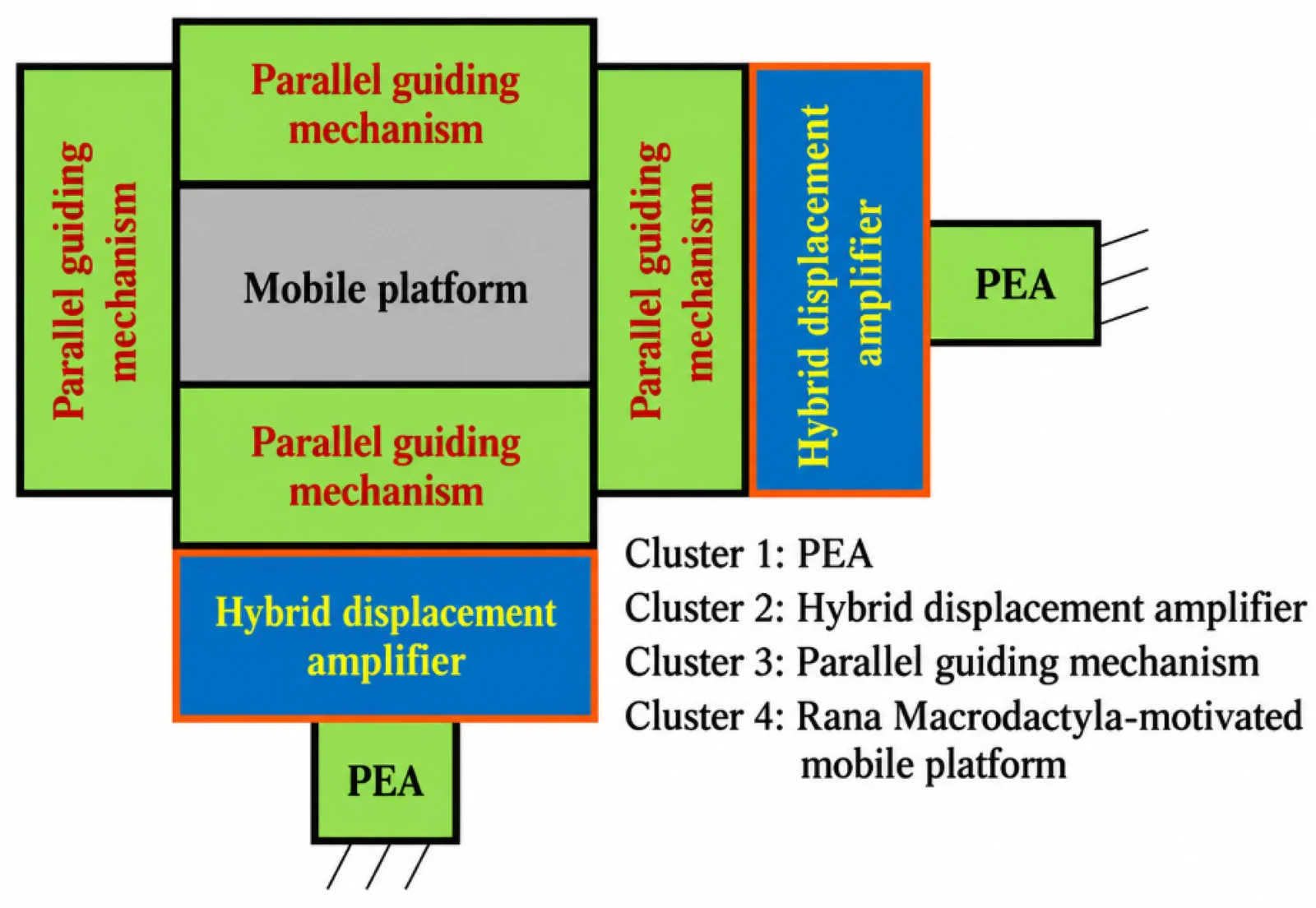
A schematic diagram of the proposed compliant positioner.

A novel XY flexure-based positioner was developed based on the flexural motion characteristics of Rana macrodactyla. It integrates a central mobile platform with a new amplification mechanism that comprises a single-lever structure and a double-rocker structure to generate linear motion in two directions and increase the working stroke. This research aims to improve the responses with a high first natural frequency, a high output displacement, and a sufficient linear working stroke. The proposed positioner, as illustrated in Figure 1, comprises the following main clusters: (i) two PEAs, (ii) two new hybrid displacement amplifiers, (iii) four parallel guiding mechanisms, and (iv) a central mobile platform.

- Cluster 1: The PEA moves the proposed structure transversely.
- Cluster 2: The hybrid displacement amplifier magnifies the PEA’s input displacement in order to increase the output displacement.
- Cluster 3: The parallel guiding mechanism based on leaf hinges reduces the parasitic motion to achieve a precise motion of the structure in one direction.
- Cluster 4: The Rana macrodactyla-inspired mobile platform is used to locate the bio-specimen for observing and checking the characteristics of the specimen.

**Figure 2:**
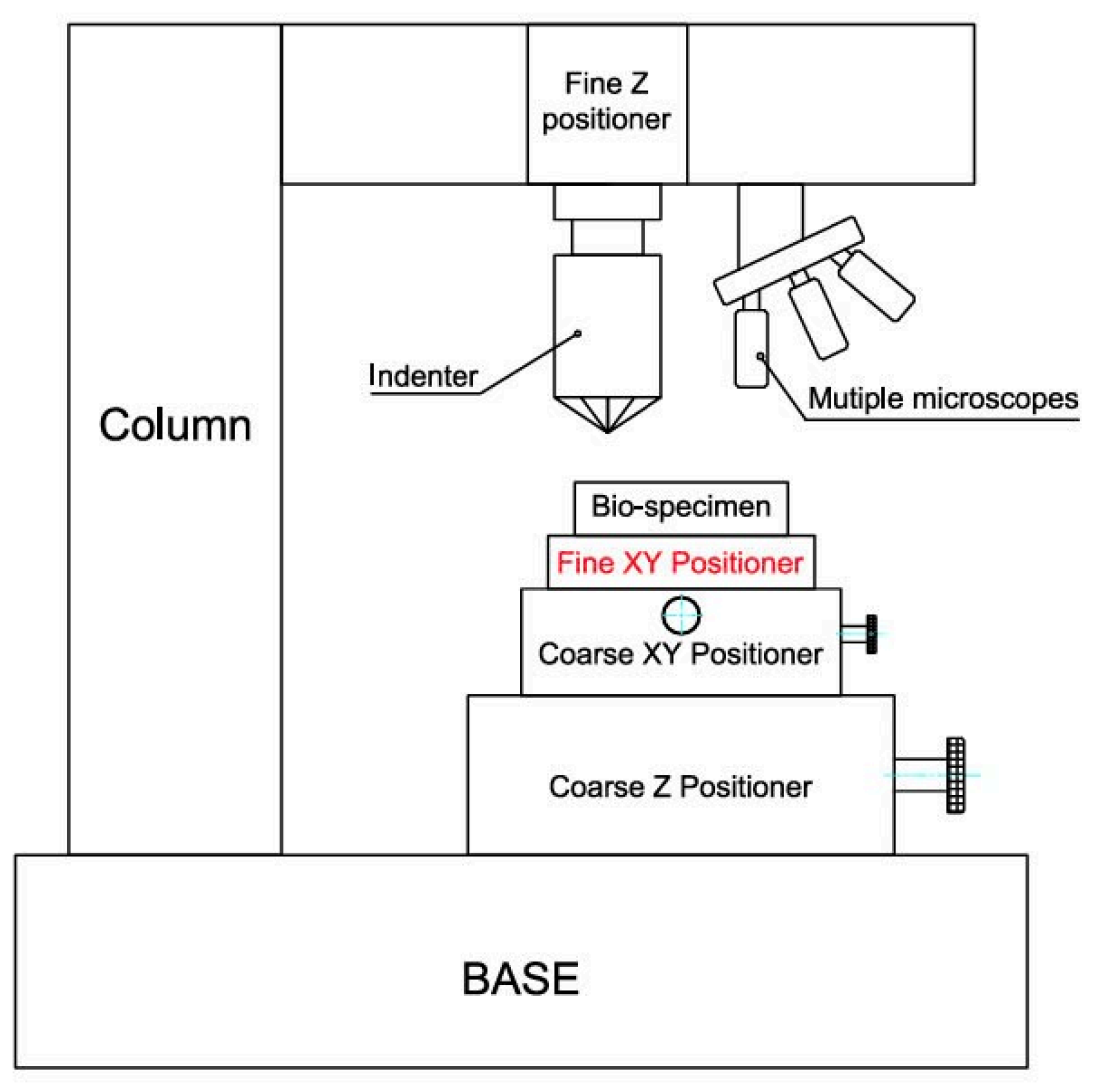
A prospective use of the proposed positioner.

A possible use of the proposed positioner in a nanoindentation testing positioning system for checking mechanical characteristics of a bio-specimen is demonstrated in Figure 2. The proposed system comprises the following main mechanisms: (i) a rough Z platform, (ii) a rough XY platform, (iii) a refined XY platform, (iv) a refined Z platform, (v) multi-microscopes, (vi) an indenter, (vii) a base, and (viii) a column.

As demonstrated in Figure 2, the operational rule of the nanoindentation testing device can be summarized in the subsequent phases. Initially, the bio-specimen is attached to the proposed positioner’s movable platform and moved in the XY direction at the microscale. In addition, the coarse Z positioner and the coarse XY positioner are utilized to move the fine XY positioner and bio-specimen to achieve a suitable location before the indentation testing process for checking the mechanical attributes of the medical specimen. The fine Z positioner positions the bio-specimen for measurement at the closest point where multi-microscopes can observe the bio-specimen’s surface. Subsequently, in order to generate an indentation on the surface of the object that is being measured, an indenter is utilized. Multi-microscopes equipped with an incorporated micro-scale range are employed to define the breadth of the indentation formed by the indenter on the surface of the specimen. As a result, the specimen’s hardness can be assessed using optical multi-microscopes. The primary aim of the suggested fine XY positioner is to generate a fine micromotion for locating the bio-specimen before the indentation testing process for evaluating the mechanical characteristics of the medical material samples.

**Figure 3:**
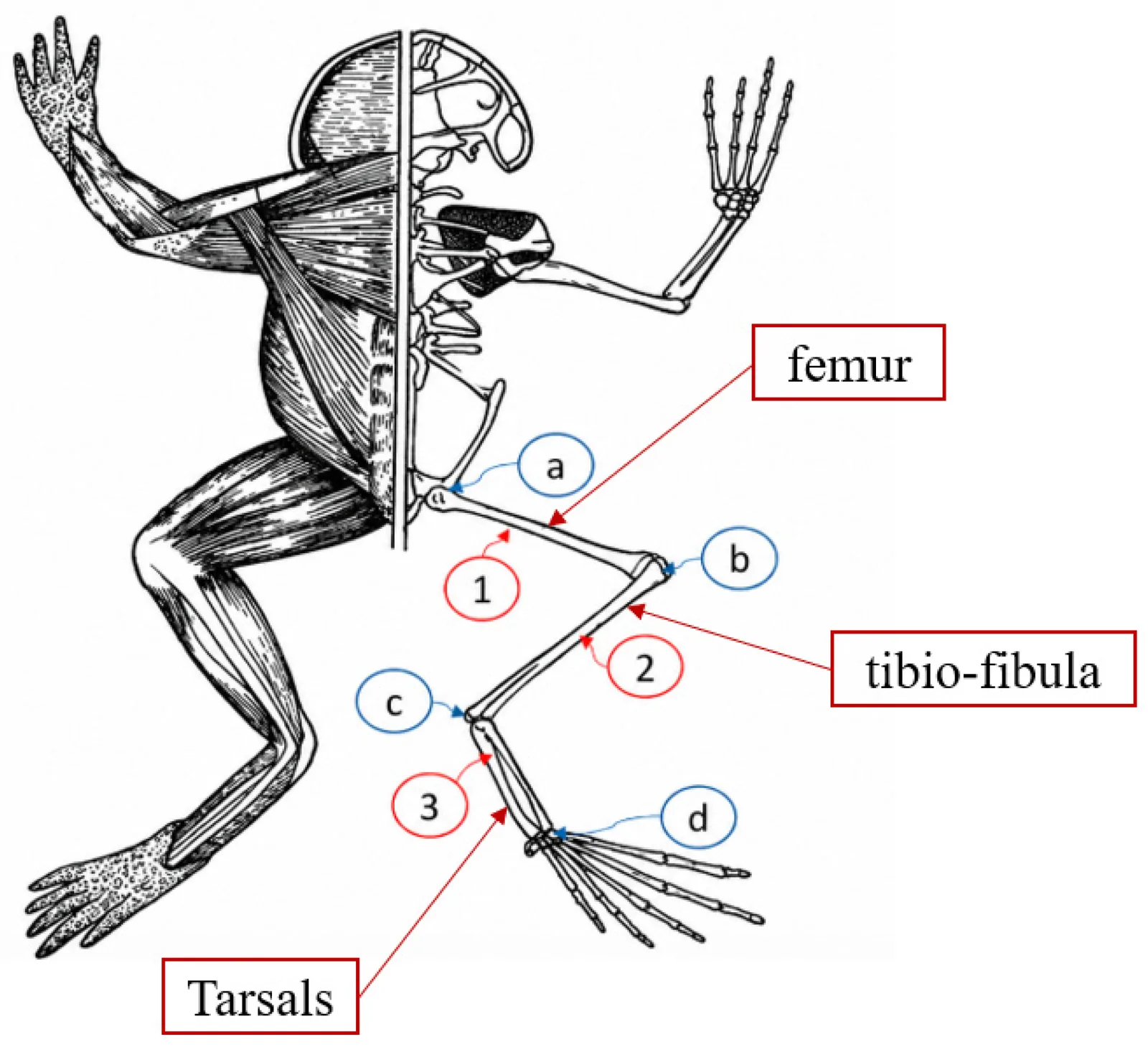
Anatomy diagram of the Rana macrodactyla.

**Figure 4:**
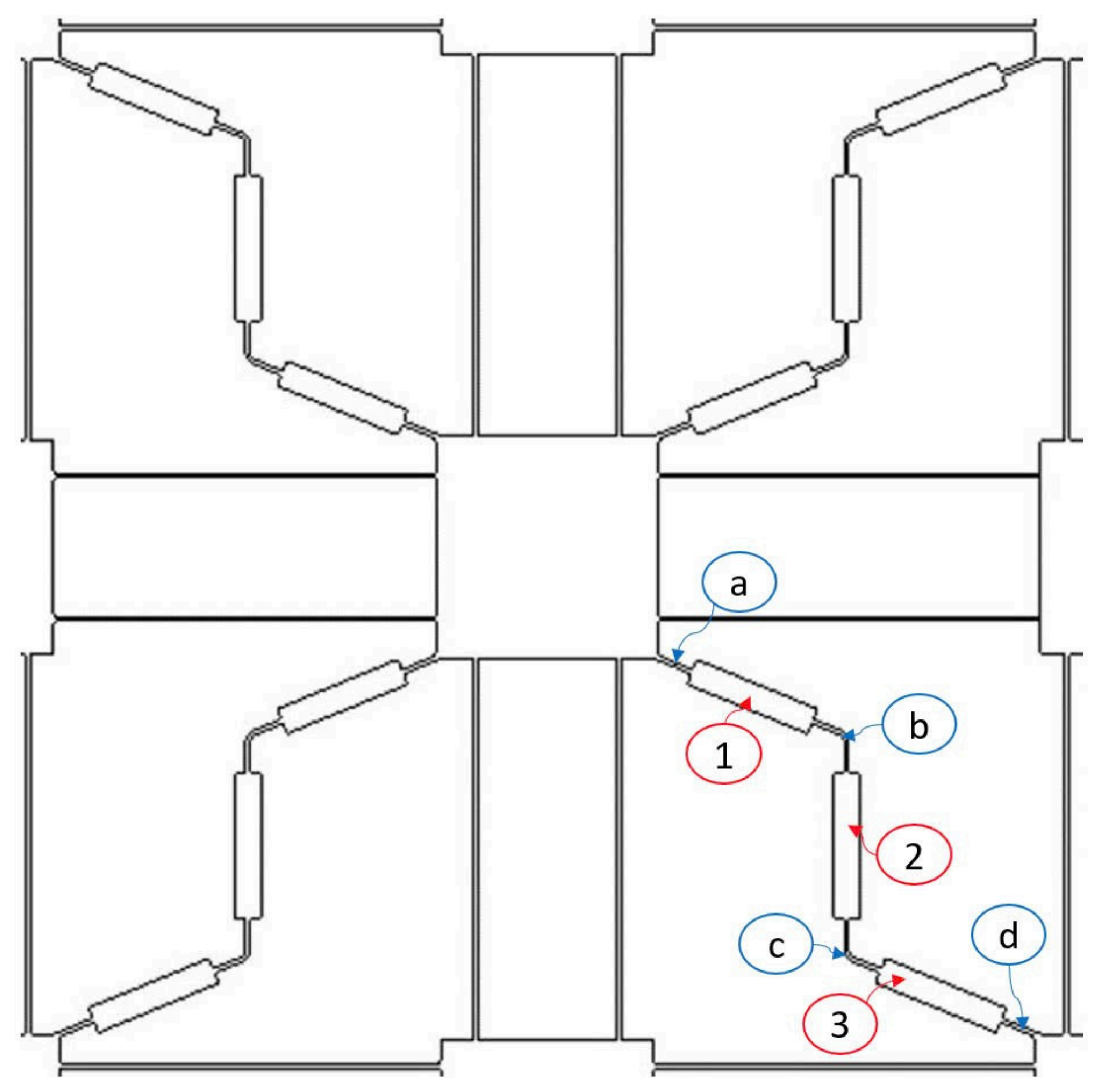
The Rana macrodactyla-inspired central mobile platform.

The developed positioner is modelled on the medical profiles and flexural movements of the Rana macrodactyla, particularly the hind-leg profile, as shown in Figure 3. The layout of the central mobile platform is also adapted based on the beneficial features of the Rana macrodactyla hind-leg profile. The Rana macrodactyla-motivated mobile platform, as illustrated in Figure 4, is supported by three symmetric rigid hinges in one direction. In each of the hind legs, there are four symmetrical flexure hinges. Therefore, the central platform can move in the X direction, Y direction, or both X-Y directions based on input displacements from PEAs.

More specifically, the proposed positioner’s biological inspiration is chiefly based on the segmented and articulated structure of the Rana macrodactyla’s hind limb rather than a direct geometric imitation of its anatomy. As depicted in Figure 3 and Figure 4, the posterior limb consists of several interlinked segments that experience synchronized relative rotations during flexion and extension. This morphological configuration was distilled into an adaptable multi-link support framework encircling the central mobile platform. The anatomical connections between adjacent limb segments were mechanically depicted by localized flexure hinges, allowing relative rotation via elastic deformation instead of traditional revolute joints. Additionally, a symmetric configuration of the relevant compliant branches was implemented to balance deformation around the central platform and enable controlled translational movement along the X and Y axes. The dimensions of the relative links, joint positions, and hinge configurations thus represent the primary technical factors that translate the biological concept into the compliant mechanism. The bio-inspired element concerns the architecture and kinematic arrangement of the mobile-platform support, whereas the hybrid lever–double-rocker amplifier is an independently designed engineering mechanism that enhances the displacement amplification ratio.

**Figure 5:**
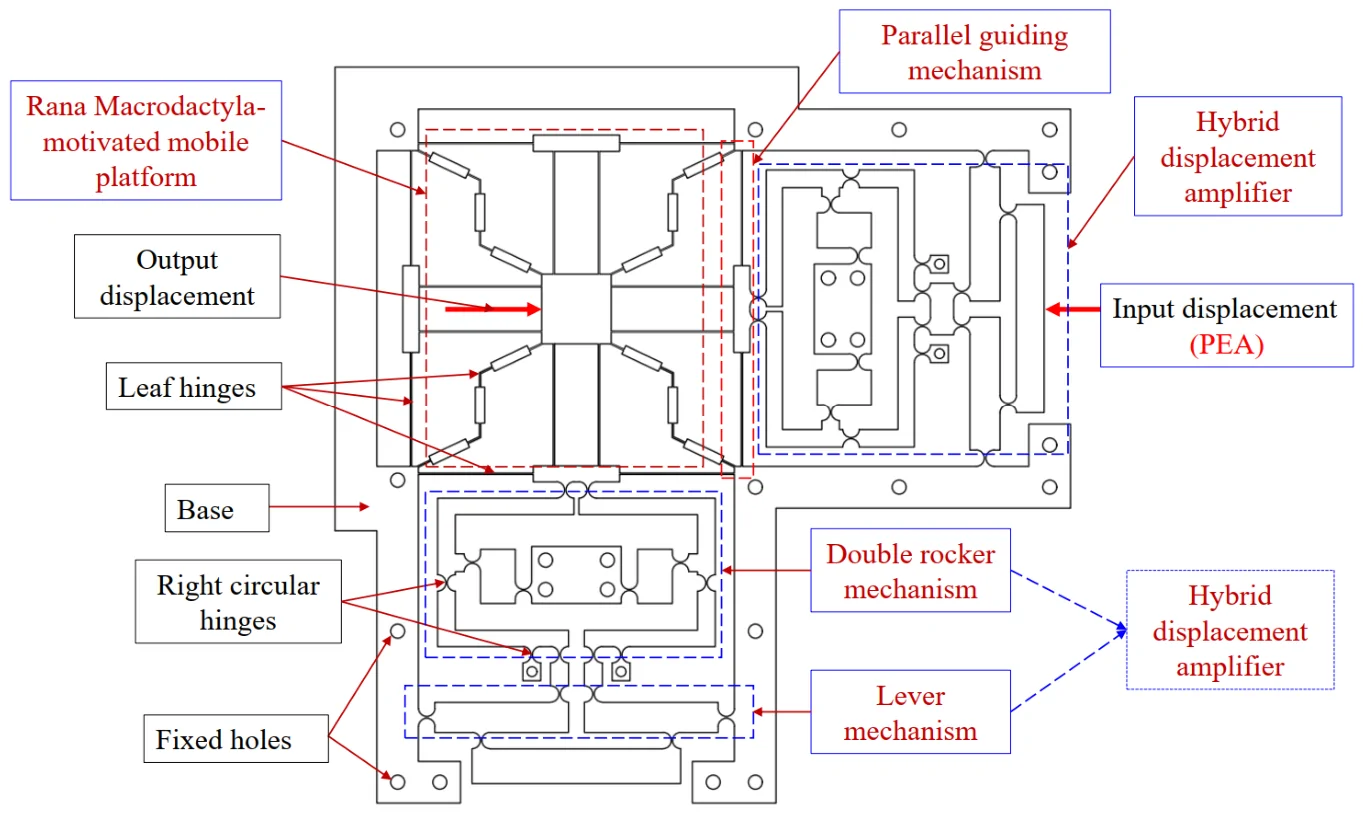
Design diagram of the developed XY positioner.

More details of the mechanical design structure are demonstrated in Figure 5. More specifically, the amplifier mechanism comprises a one-lever structure mechanism and a double-rocker structure integrated with right-circular hinges. It is designed to achieve a high displacement amplification ratio, minimal parasitic motion error, and precise control positions based on the high rotational centre accuracy of the right-circular hinges. The guiding mechanism uses rectangular hinges to reduce inaccuracies in decoupling movement. Moreover, the central mobile platform is designed based on the leg profile of the Rana macrodactyla, integrated with leaf hinges to obtain a high output displacement and reduce parasitic motion error. The material for the developed positioner is AL7075 due to its excellent performance, including an elastic modulus of 71,700 MPa, a density of 2810 kg.m^−3^, a Poisson coefficient of 0.33, and a high yield strength of 503 MPa.

**Figure 6:**
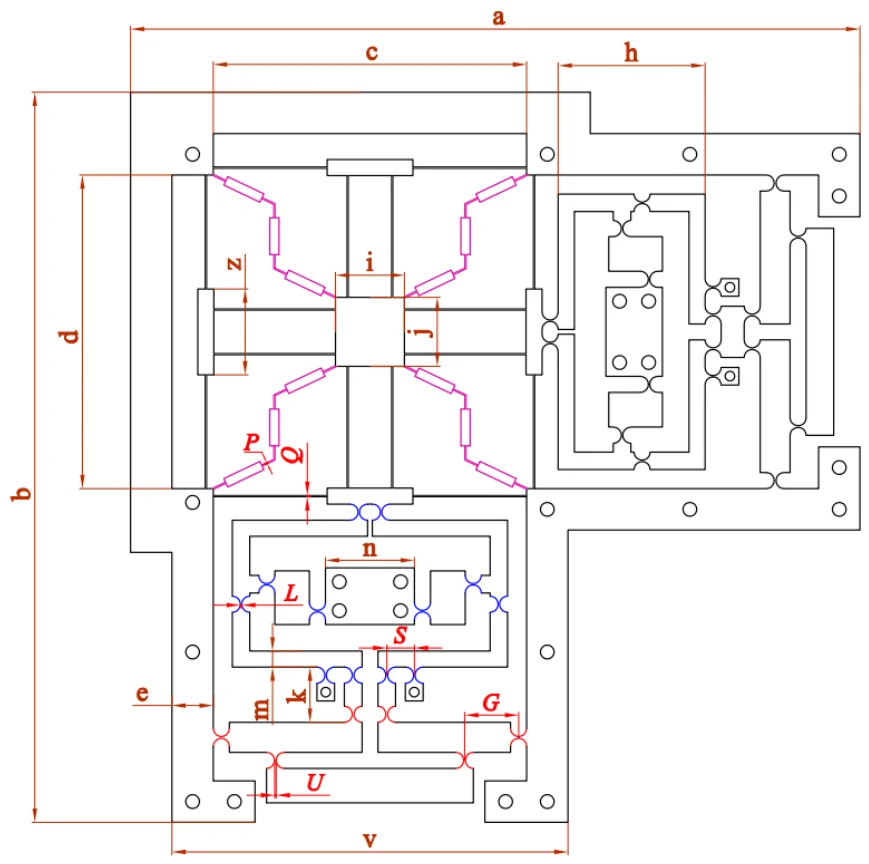
Primary main structure factors of the advanced design.

The overall dimensions of the platform are approximately 315 mm × 315 mm × 12 mm, based on the requirements of the specimen checking instrument and engineering design experience, as revealed in Figure 6. The specific values of the primary structural factors of the developed positioner are illustrated in Table 1.

**Figure 7:**
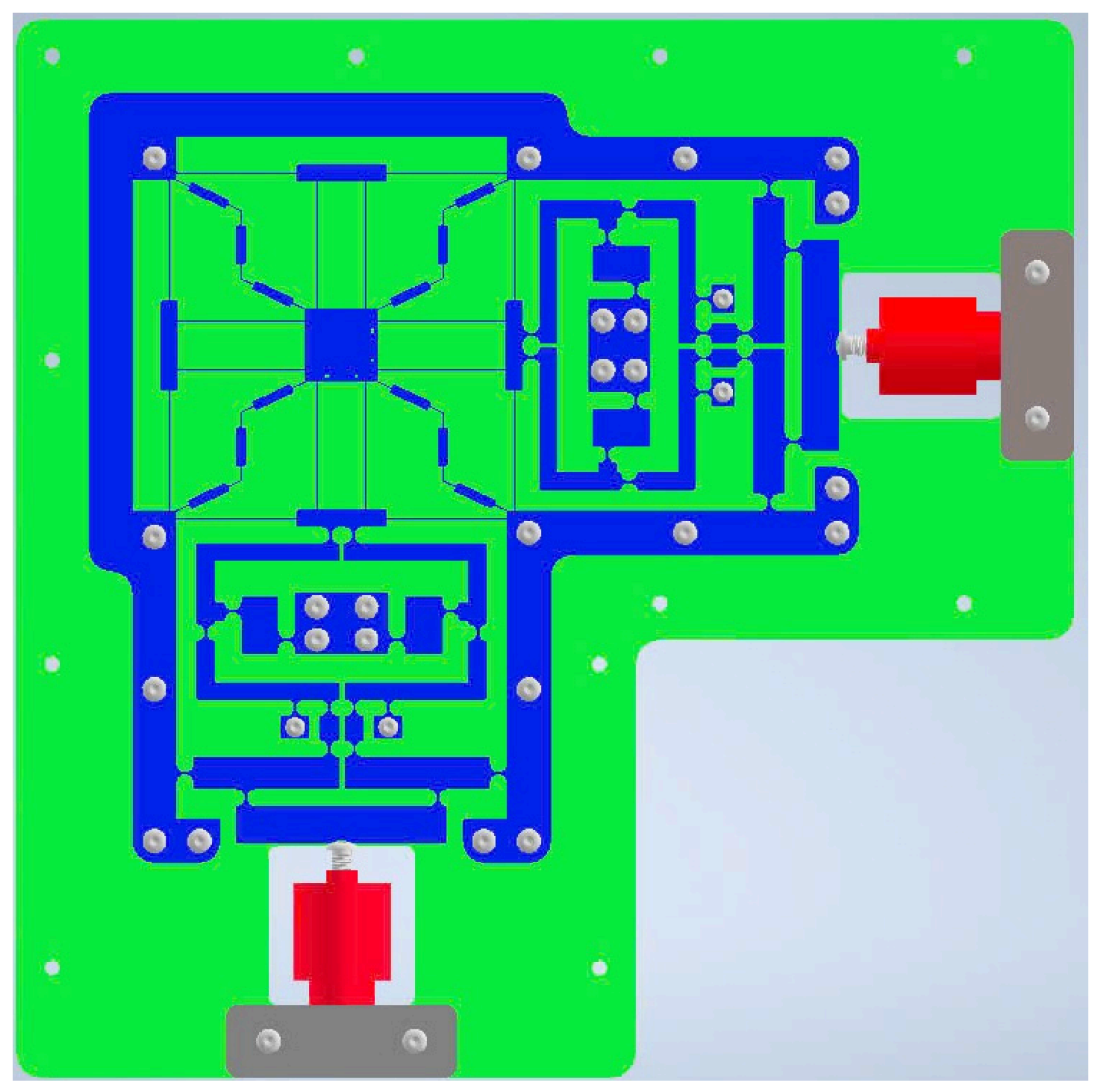
The 2D design structure.

**Figure 8:**
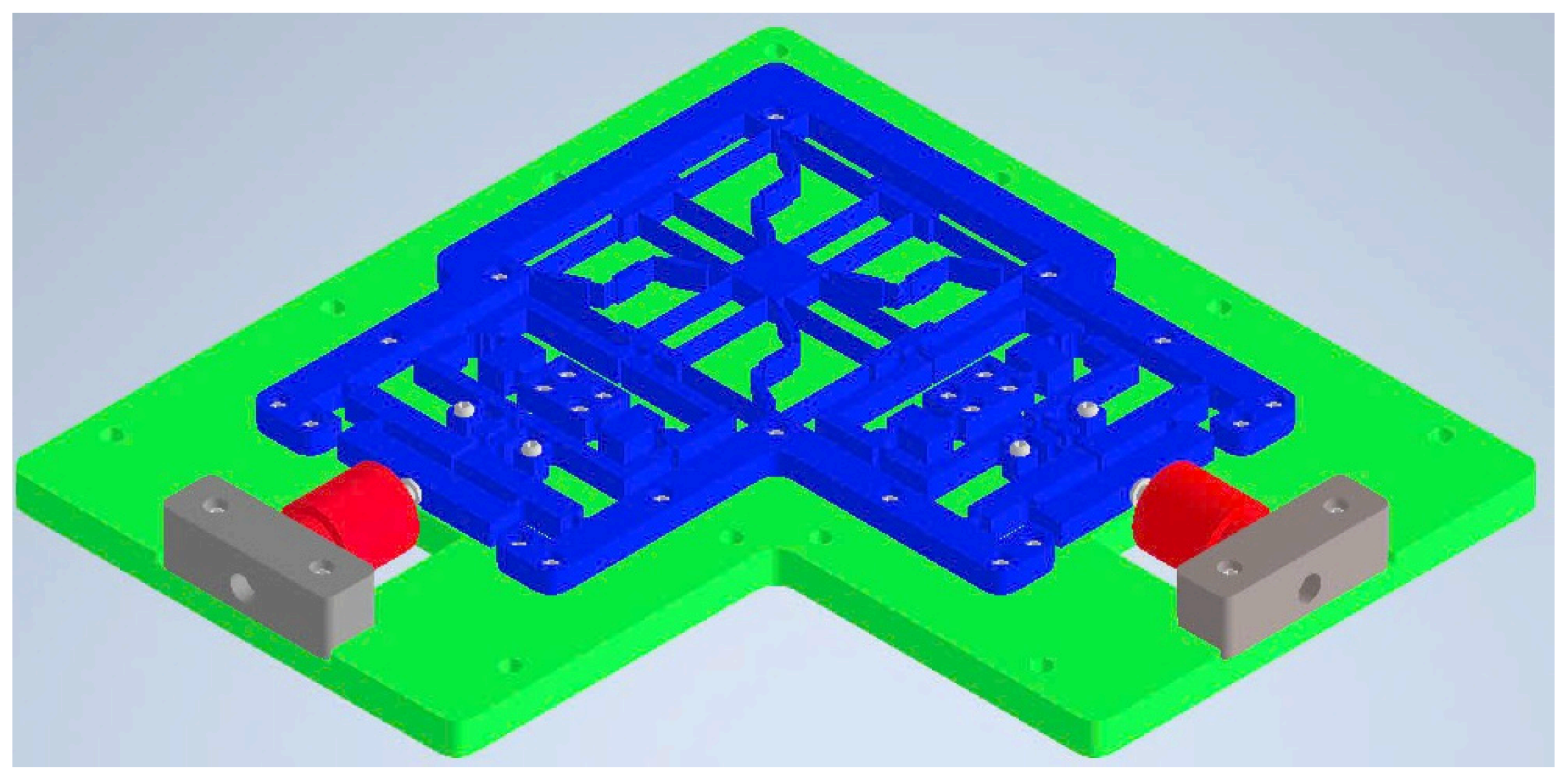
The 3D design structure.

In addition, the 2D and 3D assembly cluster design models of the proposed positioner, two PEAs, and an intermediate plate are illustrated in Figure 7 and Figure 8.

## 3. Analytical Model of the XY Positioner

### 3.1. Kinematic Analysis of the XY Positioner

**Figure 9:**
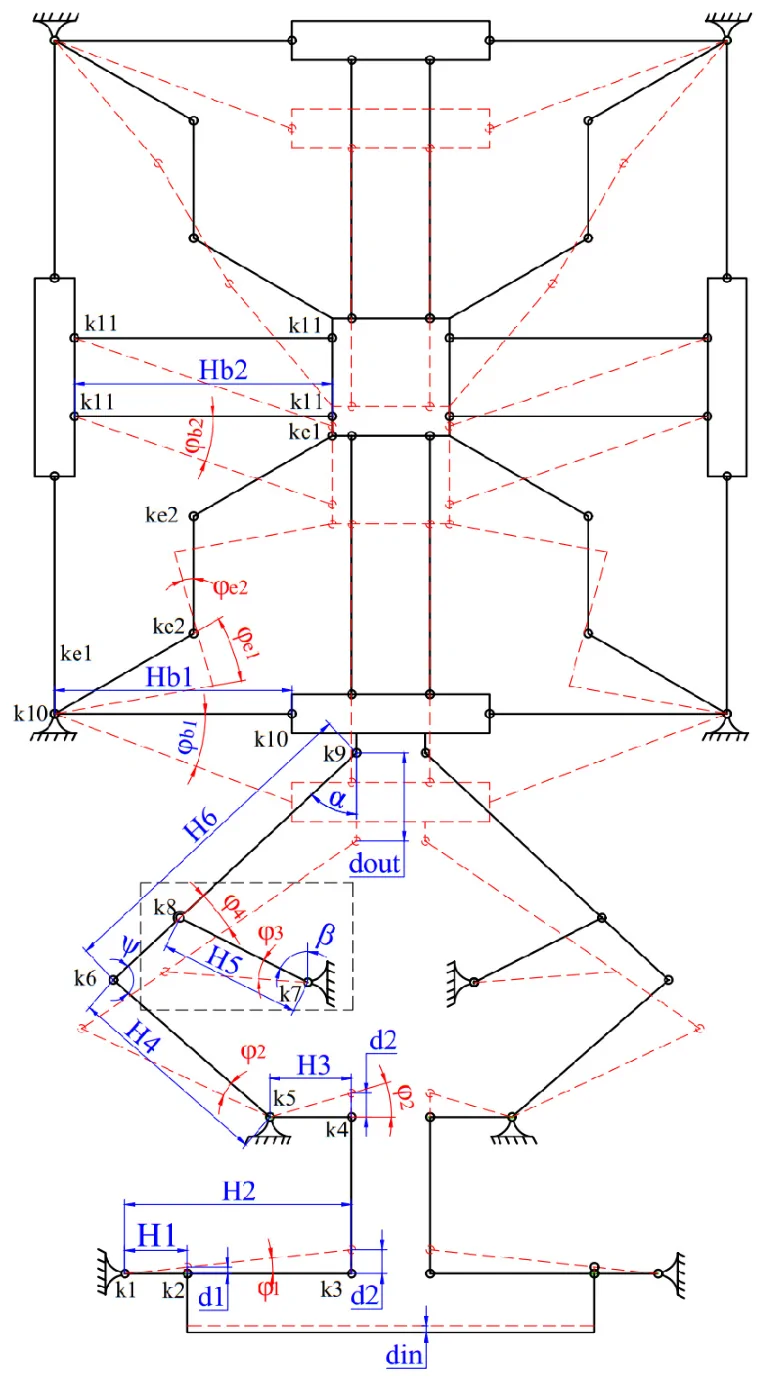
The PRBM diagram of the XY positioner.

The static and dynamic modelling for the proposed positioner was established by PRBM and the Lagrange method to assess the initial output characteristics of the developed structure rapidly. In addition, the achieved results according to the proposed analytic approach are confirmed by the FEA assessment in Ansys R19.2. The PRBM diagram of the XY positioner is demonstrated in Figure 9.

In Figure 9, the black and red edges demonstrate the positioner’s initial and deformed states under different load conditions, including no input displacement impact and an input displacement impact. The initial dimension factors for analyzing the output characteristics of the developed structure are depicted in Table 2.

**Figure 10:**
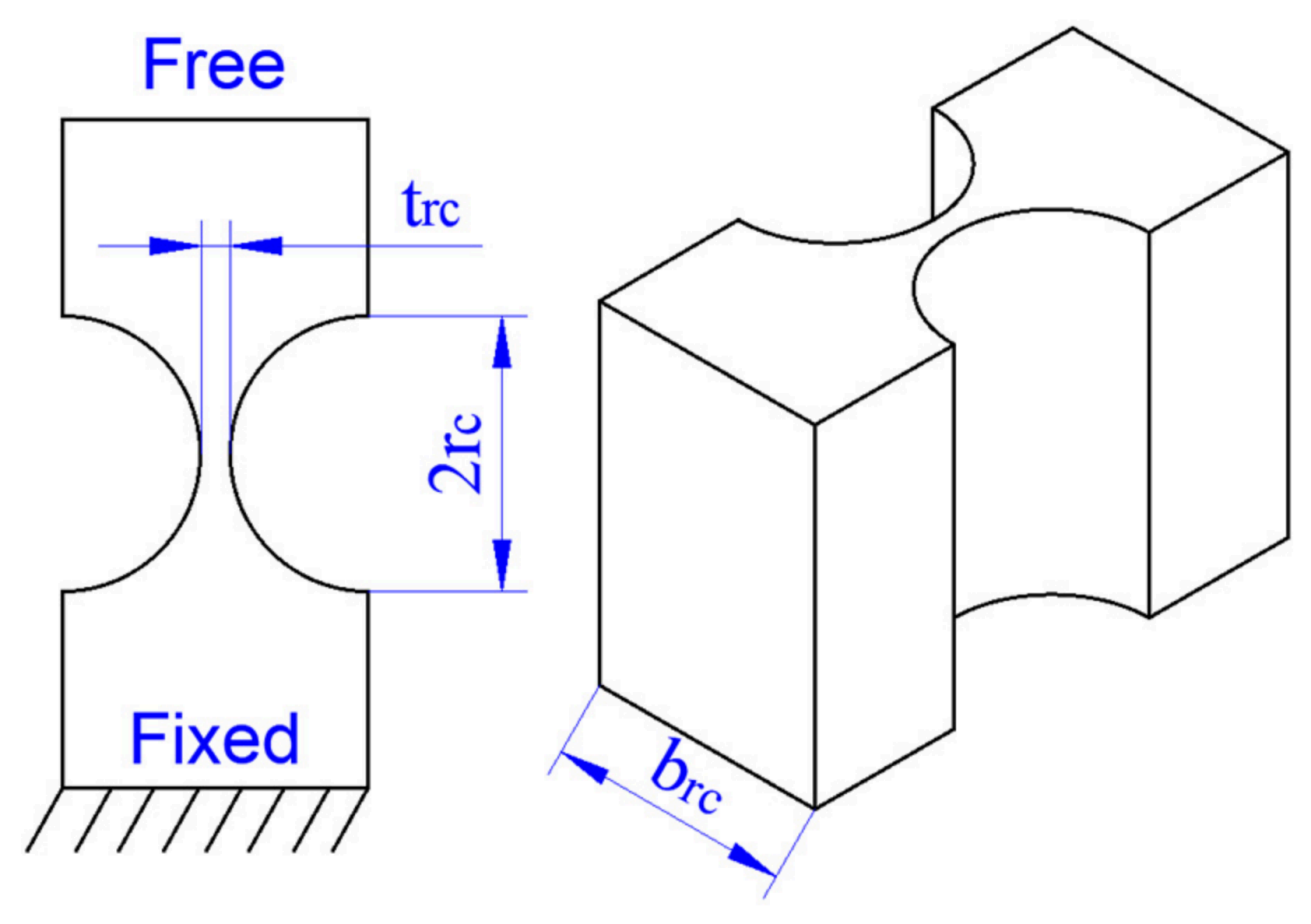
Important elements of the right-circular hinge.

**Figure 11:**
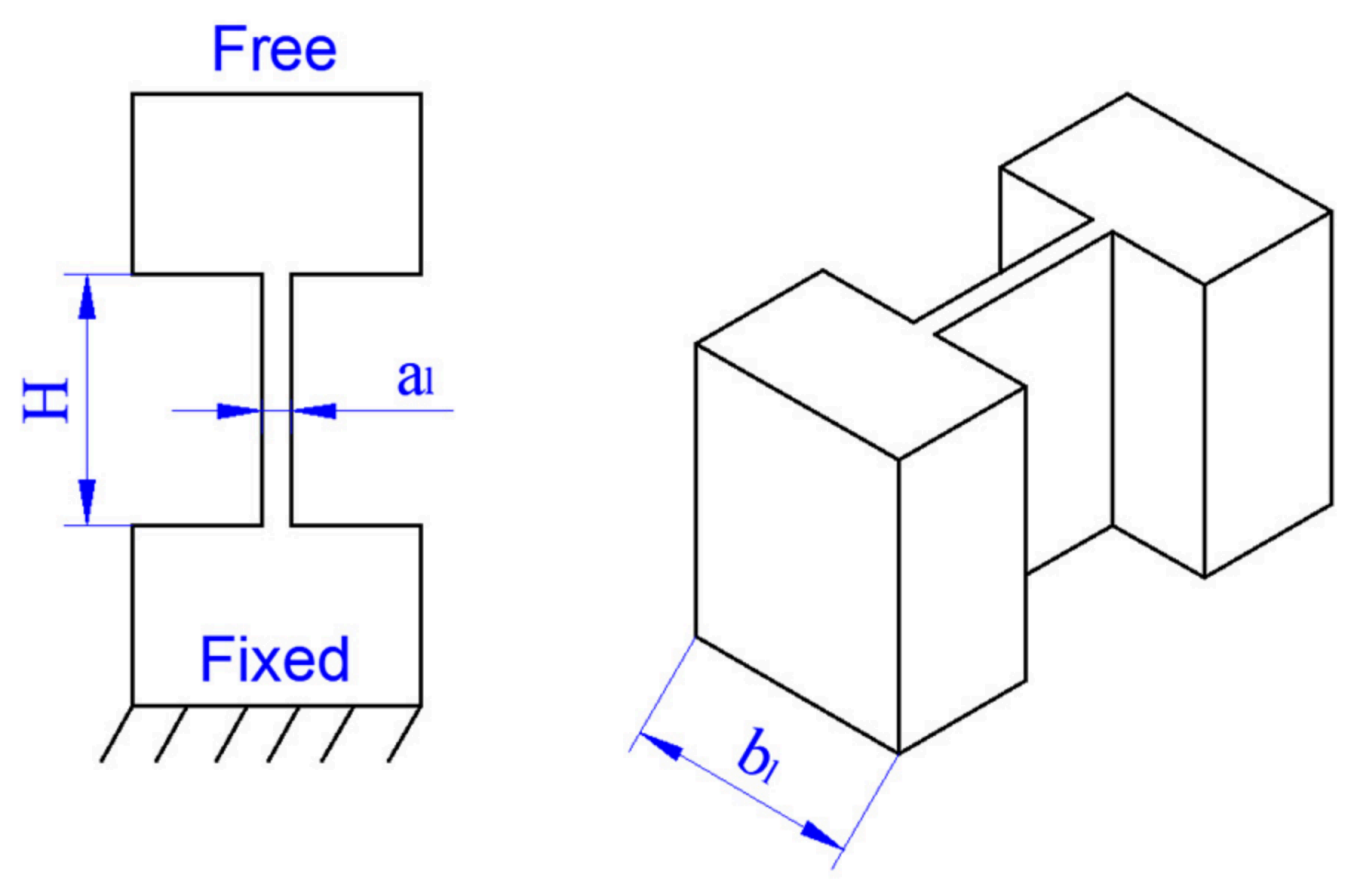
Important parameters of the leaf joint.

The right-circular hinge and leaf hinge, as demonstrated in Figure 10 and Figure 11, are utilized for developing and incorporating into the improved structure. More specifically, as portrayed in Figure 10, the main factors of the right-circular hinge comprise the thickness (*t*_rc_), the width (*b*_rc_), and the radius (*r*_c_). In addition, as demonstrated in Figure 11, the main factors of the leaf joint include the thickness (*a_l_*), the width (*b_l_*), and the length (*H*). The rotation stiffnesses of the right-circular hinge and rectangular joints are expressed in Equations (1) and (2). The inertia moment of the rotary centre at the midpoint of the bar is calculated by Equation (3), and the inertia moment of the rotary centre at the start point of the bar is described by Equation (4).

(1) $$K_{rc(j)}=\frac{2Eb_{rc(j)}t_{rc(j)}^{2.5}}{9\pi r_{c(j)}^{0.5}},\;j=1,2,\ldots \;9$$

(2) $$K_{l(n)}=\frac{Ea_{l(n)}^{3}b_{l}}{12H_{\left(n\right)}},\;n=10,\ldots \;13$$

(3) $$I_{i}=\frac{m_{i}L_{i}^{2}}{12},\;i=\mathrm{ge}$$

(4) $$I_{j}=\frac{m_{j}L_{j}^{2}}{3},\;j=1,e,b1,b2,\;H_{3},\ldots \;H_{6}$$

**Figure 12:**
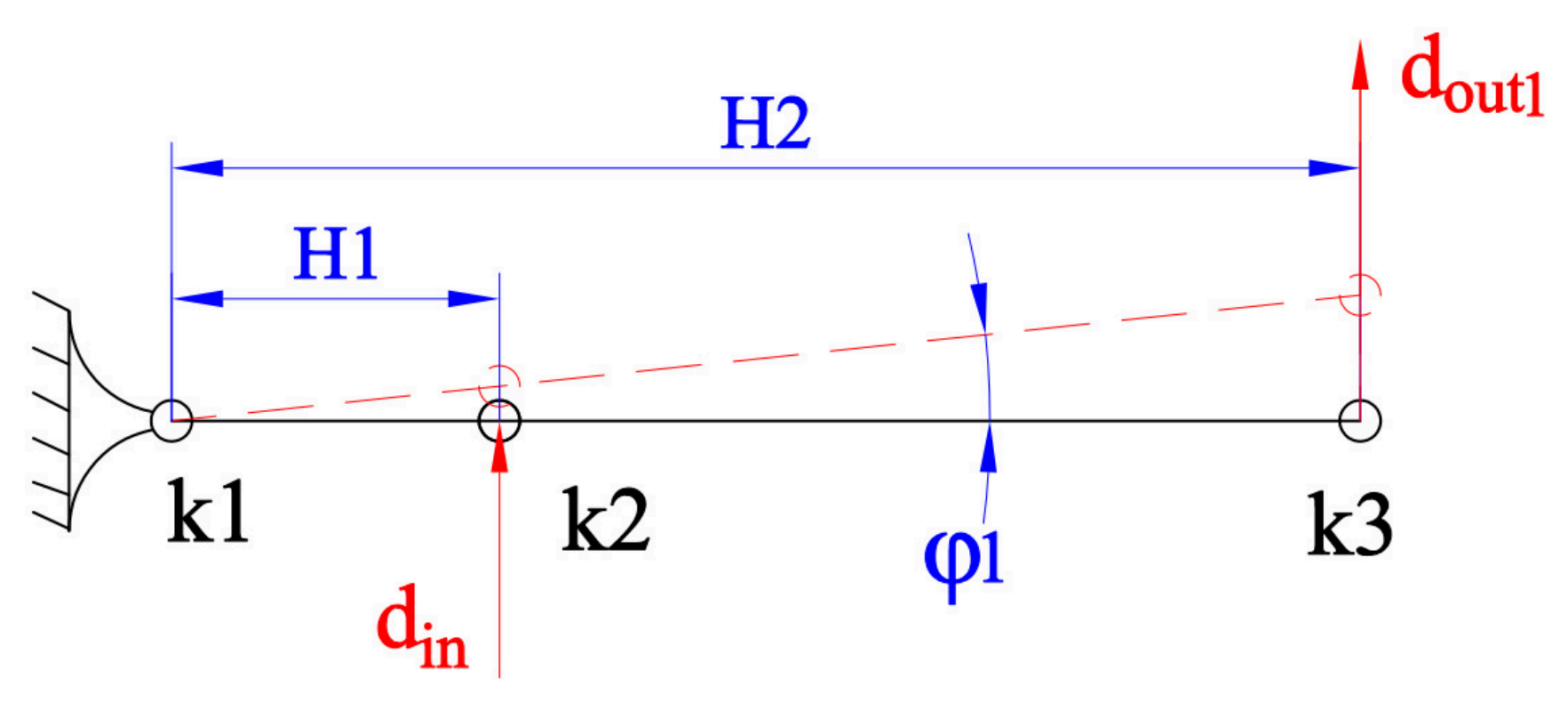
Principle scheme of the lever amplifier.

To evaluate the working stroke of the positioner, the displacement magnification ratio of the hybrid amplifier was analyzed and calculated. In addition, the displacement amplification ratios of the lever and double rocker mechanisms were analyzed. The operational rule of the lever amplifier is indicated in Figure 12.

The output displacement magnification proportion of the one-lever amplifier is defined as Equation (5).

(5) $$AR_{1}=\frac{d_{out1}}{d_{in}}=\frac{H_{2}}{H_{1}}$$

**Figure 13:**
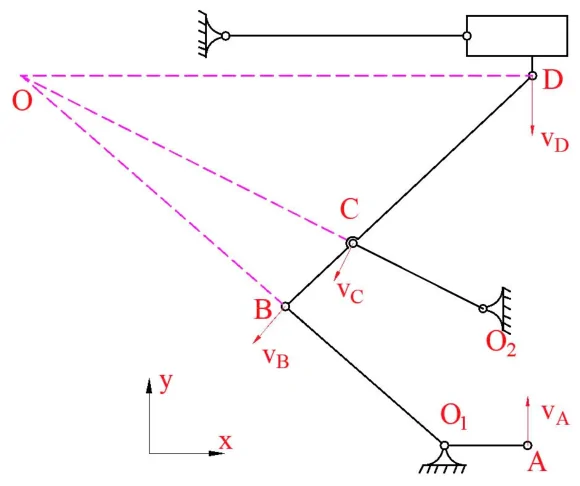
Principle diagram of the double-rocker mechanism.

**Figure 14:**
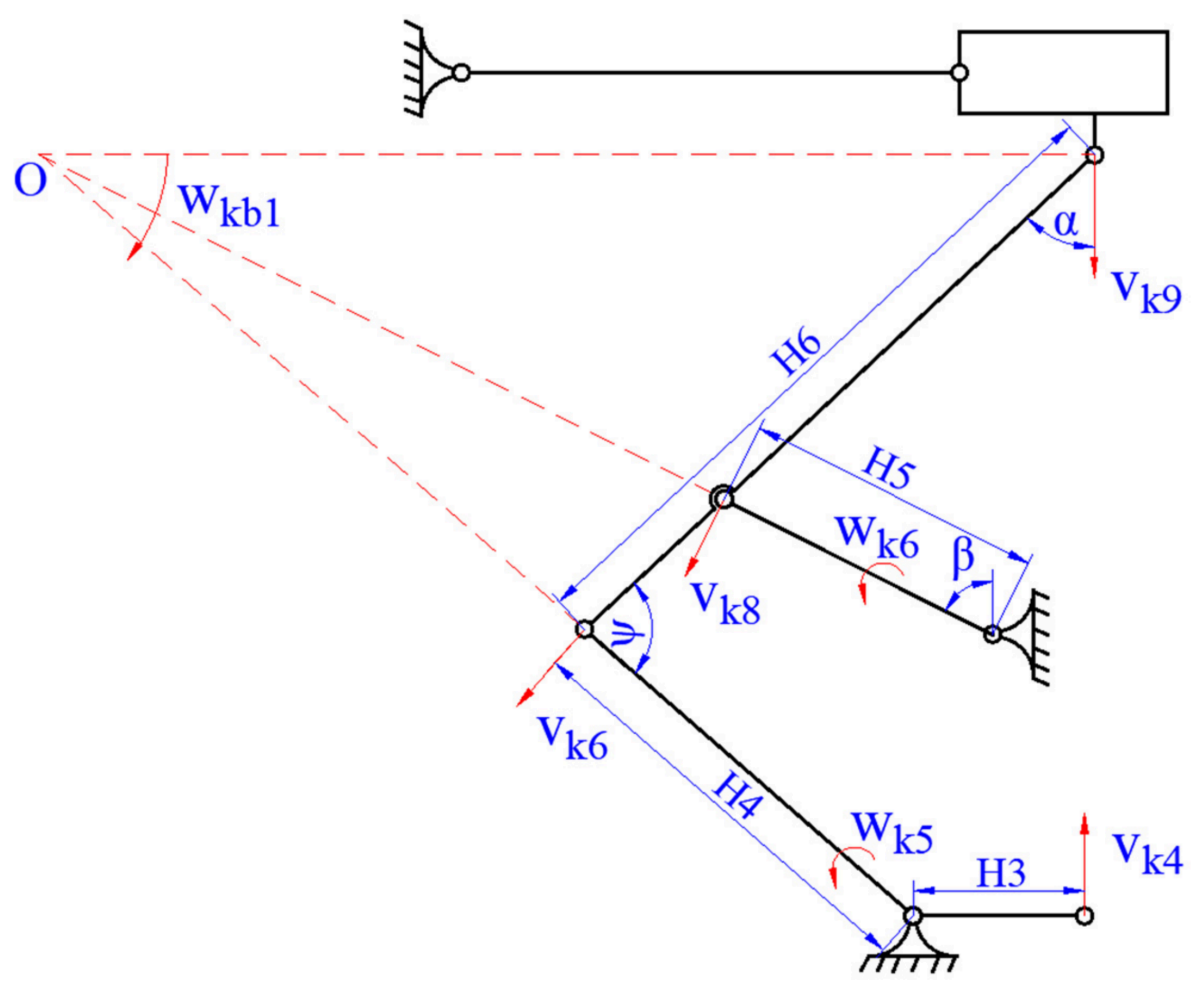
Motion vector diagram for movement in the Y direction.

In order to attain the kinematic modelling of the developed positioner, the instantaneous speed centres $O_{1},O_{2}$, and O of the links ${AO}_{1}B,O_{2}C,$ and BCD are indicated in Figure 13. Correspondingly, the immediate speeds $v_{A},\;v_{B},\;v_{C},\;v_{D}\;$at locations A, B, C, and D can be calculated by following Equations (6)–(9), respectively. In addition, the velocity vector analysis diagram of the motion points for the double rocker is demonstrated in Figure 14.

(6) $$v_{k4}=v_{A}=w_{k5}H_{3}$$

(7) $$v_{k6}=v_{B}=w_{k5}H_{4}$$

(8) $$v_{k8}=v_{C}=w_{k6}H_{5}$$

(9) $$v_{\begin{array}{l} k9 \end{array}}=v_{\begin{array}{l} D \end{array}}=w_{kb1}H_{b1}$$

(10) $$v_{k6}\cos\left(\frac{\pi }{2}-\psi \right)=v_{k8}\cos\left(\alpha +\beta -\frac{\pi }{2}\right)=v_{k9}\cos\alpha$$

Secondly, based on Equations (6)–(10), d_out_ is the output displacement ratio of the proposed double-rocker amplifier. The AR_2_ is calculated as follows:

(11) $$AR_{2}=\frac{H_{4}\cos\left(90-\psi \right)}{H_{3}\cos(\alpha )}$$

Based on Equations (5) and (11), the displacement amplification ratio of the XY positioner in one direction is calculated by the following equation:

(12) $$AR=AR_{1}\times AR_{2}=\frac{H_{2}H_{4}\cos\left(90-\psi \right)}{H_{1}H_{3}\cos(\alpha )}$$

### 3.2. Dynamic Analysis of the Proposed Positioner

Let $\varphi_{j}$ (*j* = 1, 2… 4, $b_{1}$, $b_{2}$, *e*, g*e*) represent the altered angles of the related connections subjected to an input impact. The correlations between the rotation angles were then computed using the equations listed below.

(13) $$\varphi_{1}\approx \frac{d_{1}}{H_{1}}$$

(14) $$\varphi_{2}=\frac{H_{3}}{H_{2}}\varphi_{1}$$

(15) $$\varphi_{3}=\frac{H_{2}H_{4}}{H_{3}H_{5}}\times \frac{\cos\left(90-\psi \right)}{\cos\left(\alpha +\beta -90\right)}\times \varphi_{1}$$

(16) $$\varphi_{4}=\frac{H_{2}H_{4}}{H_{3}H_{6}}\times \frac{\sin\left(\alpha +\psi -90\right)}{\sin\left(90-\alpha \right)}\times \varphi_{1}$$

(17) $$\varphi_{b1}=\frac{d_{out}}{H_{b1}}=\frac{H_{2}H_{4}\cos\left(90-\psi \right)}{H_{1}H_{3}\cos(\alpha )}\times \frac{d_{in}}{H_{b1}}=\frac{H_{2}H_{4}\cos\left(90-\psi \right)}{H_{3}\cos(\alpha )H_{b1}}\times \varphi_{1}$$

(18) $$\varphi_{b2}=\frac{d_{out}}{H_{b2}}=\frac{H_{2}H_{4}\cos\left(90-\psi \right)}{H_{1}H_{3}\cos(\alpha )}\times \frac{d_{in}}{H_{b2}}=\frac{H_{2}H_{4}\cos\left(90-\psi \right)}{H_{3}\cos(\alpha )H_{b2}}\times \varphi_{1}$$

**Figure 15:**
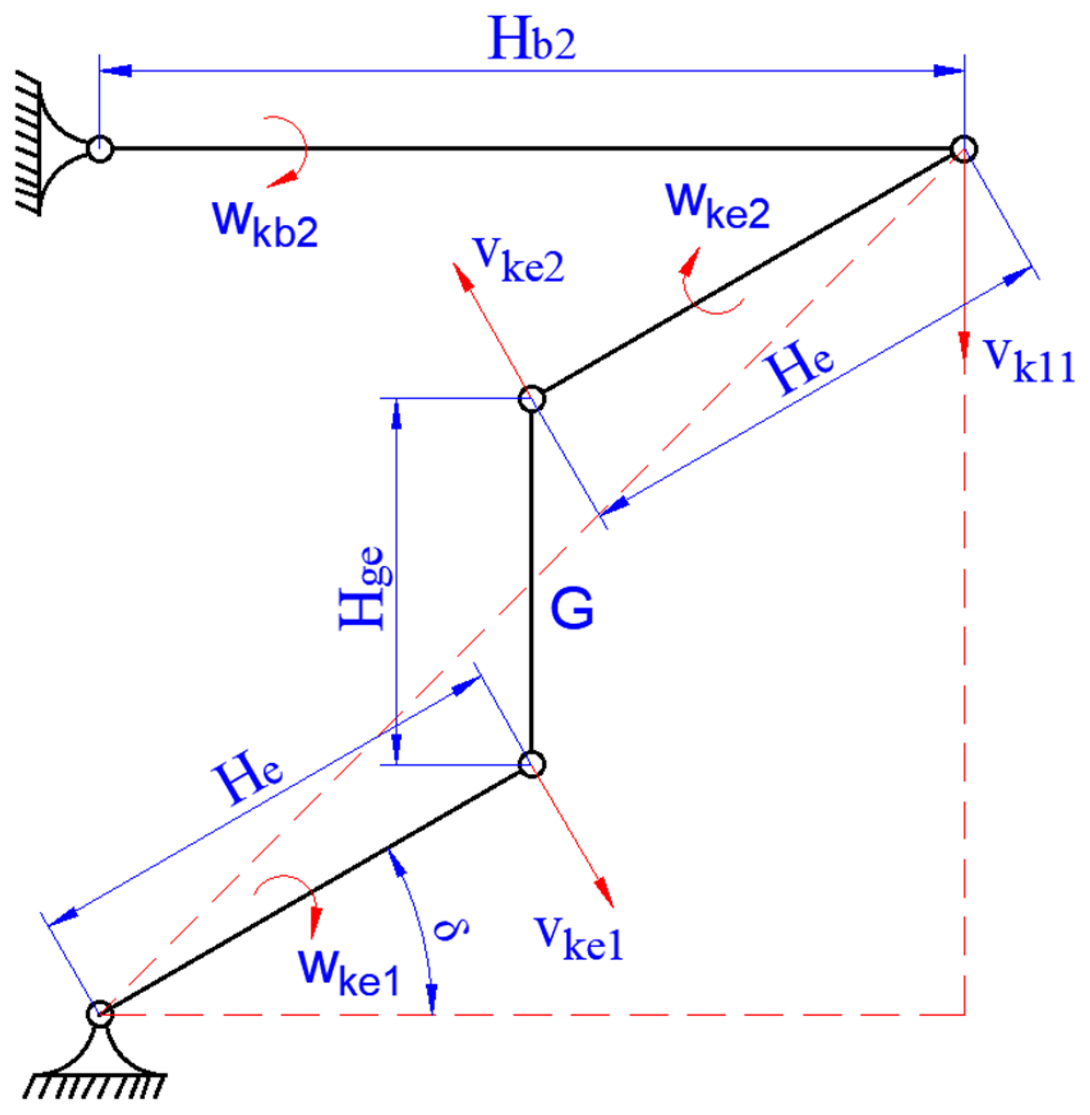
Motion vector diagram for Y-directional motion.

The motion vector diagram for the movement of the Rana macrodactyla-inspired structure in the Y direction is illustrated in Figure 15.

Based on the velocity analysis diagram, as illustrated in Figure 15, the instantaneous velocities v_k11_, v_ke1_, and v_ke2_ can be expressed as follows:

(19) $$v_{k11}=w_{b2}H_{b2}$$

(20) $$v_{ke1}=w_{ke1}H_{e}$$

(21) $$v_{ke2}=w_{ke2}H_{e}$$

(22) $$v_{ke1}=v_{ke2}=\frac{1}{2}\times v_{k11}\cos(90-\delta )$$

The φ_e_ and φ_ge_ rotation angle distortions are represented as follows:

(23) $$\varphi_{e}=\frac{H_{b2}\cos\left(90-\delta \right)}{2H_{e}}\times \frac{H_{2}H_{4}\cos\left(90-\psi \right)}{H_{3}\cos(\alpha )H_{b2}}\times \varphi_{1}$$

(24) $$\varphi_{ge}=\frac{H_{b2}\cos\left(\delta \right)}{2H_{ge}}\times \frac{H_{2}H_{4}\cos\left(90-\psi \right)}{H_{3}\cos(\alpha )H_{b2}}\times \varphi_{1}$$

The kinetic energy of the proposed one-direction structure is identified as follows:

(25) $$E_{K}=\sum_{1}^{n}\left(E_{t}{+E}_{r}\right)=\sum_{1}^{n}\left(\frac{1}{2}m_{i}v_{i}^{2}{+I}_{i}\varphi_{j}^{2}\right)$$

(26) $$\begin{array}{l} E_{K}=\frac{1}{2}m_{1}{\left(\frac{H_{2}}{2}{\dot{\varphi }}_{1}\right)}^{2}+\frac{1}{2}I_{1}{\dot{\varphi }}_{1}^{2}+\frac{1}{2}m_{a}{\left(\frac{H_{2}}{2}{\dot{\varphi }}_{1}\right)}^{2}+\frac{1}{2}m_{H_{3}}{\left(\frac{H_{3}}{2}{\dot{\varphi }}_{2}\right)}^{2}+\frac{1}{2}I_{H_{3}}{\dot{\varphi }}_{2}^{2}\\ +\frac{1}{2}m_{H_{4}}{\left(\frac{H_{4}}{2}{\dot{\varphi }}_{2}\right)}^{2}+\frac{1}{2}I_{H_{4}}{\dot{\varphi }}_{2}^{2}+\frac{1}{2}m_{H_{5}}{\left(\frac{H_{5}}{2}{\dot{\varphi }}_{3}\right)}^{2}+\frac{1}{2}I_{H_{5}}{\dot{\varphi }}_{3}^{2}+\frac{1}{2}m_{H_{6}}{\left(\frac{H_{6}}{2}{\dot{\varphi }}_{4}\right)}^{2}\\ +\frac{1}{2}I_{H_{6}}{\dot{\varphi }}_{4}^{2}+m_{H_{b1}}{\left(\frac{H_{b1}}{2}{\dot{\varphi }}_{b1}\right)}^{2}+I_{H_{b1}}{\dot{\varphi }}_{b1}^{2}+m_{H_{b2}}{\left(\frac{H_{b2}}{2}{\dot{\varphi }}_{b2}\right)}^{2}+I_{H_{b2}}{\dot{\varphi }}_{b2}^{2}\\ +\frac{1}{4}m_{1a}{\left(\frac{H_{b1}}{2}{\dot{\varphi }}_{bl1}\right)}^{2}+m_{2a}{\left(\frac{H_{b1}}{2}{\dot{\varphi }}_{bl1}\right)}^{2}+2m_{e}{\left(\frac{H_{e}}{2}{\dot{\varphi }}_{e}\right)}^{2}+m_{ge}{\left(\frac{H_{e}}{2}{\dot{\varphi }}_{ge}\right)}^{2} \end{array}$$

where the symbols $E_{K}$, $E_{t}$, and $E_{r}$ represent the kinetic energy, the linear kinetic energy, and the rotational kinetic energy, respectively.

In addition, the elastic potential energy of the proposed structure cluster is identified by Equation (27).

(27) $$E_{V}=\sum_{I=1}^{12}\left(\frac{1}{2}K_{j}\varphi_{j}^{2}\right)$$

(28) $$\begin{array}{l} E_{V}=\frac{1}{2}\left(k_{1}+k_{2}+k_{3}\right)\varphi_{1}^{2}+\frac{1}{2}\left(k_{4}+k_{5}\right)\varphi_{2}^{2}+\frac{1}{2}k_{7}\varphi_{3}^{2}+\frac{1}{2}k_{6}{\left(\varphi_{2}+\varphi_{4}\right)}^{2}+\frac{1}{2}k_{8}{\left(\varphi_{3}+\varphi_{4}\right)}^{2}\\ +\frac{1}{2}k_{9}\varphi_{4}^{2}+4\times \frac{1}{2}k_{b1}\varphi_{b1}^{2}+4\times \frac{1}{2}k_{b2}\varphi_{b2}^{2}+4\times \frac{1}{2}k_{e1}\varphi_{e}^{2}+4\times \frac{1}{2}k_{e2}\varphi_{ge}^{2} \end{array}$$

where *E_v_* denotes the system’s potential energy, *k_j_* signifies the spring stiffness in the proposed structure, and *ϕ_j_* symbolizes the rotation angle in the developed structure.

Replacing Equations (13)–(18), (23), and (24) into Equation (28), the *E_v_* is calculated as follows:

(29) $$\begin{array}{l} E_{V}=\left[\frac{1}{2}\left(k_{1}+k_{2}+k_{3}\right)+\frac{1}{2}\left(k_{4}+k_{5}\right){\left(\frac{H_{3}}{H_{2}}\right)}^{2}+\frac{1}{2}k_{7}{\left(\frac{H_{2}H_{4}}{H_{3}H_{5}}\times \frac{\cos\left(90-\psi \right)}{\cos\left(\alpha +\beta -90\right)}\right)}^{2}\right.\\ +\frac{1}{2}k_{6}{\left(\frac{H_{3}}{H_{2}}+\frac{H_{2}H_{4}}{H_{3}H_{6}}\times \frac{\sin\left(\alpha +\psi -90\right)}{\sin\left(90-\alpha \right)}\right)}^{2}+\frac{1}{2}k_{8}{\left(\frac{H_{2}H_{4}}{H_{3}H_{5}}\times \frac{\cos\left(90-\psi \right)}{\cos\left(\alpha +\beta -90\right)}+\frac{H_{2}H_{4}}{H_{3}H_{6}}\times \frac{\sin\left(\alpha +\psi -90\right)}{\sin\left(90-\alpha \right)}\right)}^{2}\\ +\frac{1}{2}k_{9}{\left(\frac{H_{2}H_{4}}{H_{3}H_{6}}\times \frac{\sin\left(\alpha +\psi -90\right)}{\sin\left(90-\alpha \right)}\right)}^{2}+2k_{b1}{\left(\frac{H_{2}H_{4}\cos\left(90-\psi \right)}{H_{3}\cos(\alpha )H_{b1}}\right)}^{2}+2k_{b2}{\left(\frac{H_{2}H_{4}\cos\left(90-\psi \right)}{H_{3}\cos(\alpha )H_{b2}}\right)}^{2}\\ \left.+2k_{e1}{\left(\frac{H_{b2}\cos\left(90-\delta \right)}{2H_{e}}\times \frac{H_{2}H_{4}\cos\left(90-\psi \right)}{H_{b2}H_{3}\cos\left(\alpha \right)}\right)}^{2}+2k_{e2}{\left(\frac{H_{b2}\cos\left(\delta \right)}{2H_{ge}}\times \frac{H_{2}H_{4}\cos\left(90-\psi \right)}{H_{b2}H_{3}\cos\left(\alpha \right)}\right)}^{2}\right]\varphi_{1}^{2} \end{array}$$

Replacing Equation (13) into Equation (29), *E_v_* is expressed as follows:

(30) $$\begin{array}{l} E_{V}=\left[\frac{1}{2}\left(k_{1}+k_{2}+k_{3}\right){\left(\frac{1}{H_{1}}\right)}^{2}+\frac{1}{2}\left(k_{4}+k_{5}\right){\left(\frac{H_{3}}{H_{2}H_{1}}\right)}^{2}+\frac{1}{2}k_{7}{\left(\frac{H_{2}H_{4}}{H_{1}H_{3}H_{5}}\times \frac{\cos\left(90-\psi \right)}{\cos\left(\alpha +\beta -90\right)}\right)}^{2}\right.\\ +\frac{1}{2}k_{6}{\left(\frac{H_{3}}{H_{2}}+\frac{H_{2}H_{4}}{H_{3}H_{6}}\times \frac{\sin\left(\alpha +\psi -90\right)}{\sin\left(90-\alpha \right)}\right)}^{2}{\left(\frac{1}{H_{1}}\right)}^{2}+\frac{1}{2}k_{8}{\left(\frac{H_{2}H_{4}}{H_{3}H_{5}}\times \frac{\cos\left(90-\psi \right)}{\cos\left(\alpha +\beta -90\right)}+\frac{H_{2}H_{4}}{H_{3}H_{6}}\times \frac{\sin\left(\alpha +\psi -90\right)}{\sin\left(90-\alpha \right)}\right)}^{2}{\left(\frac{1}{H_{1}}\right)}^{2}\\ +\frac{1}{2}k_{9}{\left(\frac{H_{2}H_{4}}{H_{3}H_{6}}\times \frac{\sin\left(\alpha +\psi -90\right)}{\sin\left(90-\alpha \right)}\right)}^{2}{\left(\frac{1}{H_{1}}\right)}^{2}+2k_{b1}{\left(\frac{H_{2}H_{4}\cos\left(90-\psi \right)}{H_{3}\cos(\alpha )H_{b1}}\right)}^{2}{\left(\frac{1}{H_{1}}\right)}^{2}+2k_{b2}{\left(\frac{H_{2}H_{4}\cos\left(90-\psi \right)}{H_{3}\cos(\alpha )H_{b2}}\right)}^{2}{\left(\frac{1}{H_{1}}\right)}^{2}\\ \left.+2k_{e1}{\left(\frac{H_{b2}\cos\left(90-\delta \right)}{2H_{e}}\times \frac{H_{2}H_{4}\cos\left(90-\psi \right)}{H_{b2}H_{3}\cos\left(\alpha \right)}\right)}^{2}{\left(\frac{1}{H_{1}}\right)}^{2}+2k_{e2}{\left(\frac{H_{b2}\cos\left(\delta \right)}{2H_{ge}}\times \frac{H_{2}H_{4}\cos\left(90-\psi \right)}{H_{b2}H_{3}\cos\left(\alpha \right)}\right)}^{2}{\left(\frac{1}{H_{1}}\right)}^{2}\right]d_{in}^{2} \end{array}$$

The work W performed by the force operating on the amplification mechanism is computed as follows:

(31) $$W=\frac{1}{2}F_{in}d_{in}$$

In accordance with the energy conservation principle, the work is entirely transformed into the elastic potential energy of the spring. Therefore, W = E_V_, and the equivalent balance formula is established as follows:

(32) $$\begin{array}{l} F_{in}d_{in}=\left[\left(k_{1}+k_{2}+k_{3}\right){\left(\frac{1}{H_{1}}\right)}^{2}+\left(k_{4}+k_{5}\right){\left(\frac{H_{3}}{H_{2}H_{1}}\right)}^{2}+k_{7}{\left(\frac{H_{2}H_{4}}{H_{1}H_{3}H_{5}}\times \frac{\cos\left(90-\psi \right)}{\cos\left(\alpha +\beta -90\right)}\right)}^{2}\right.\\ +k_{6}{\left(\frac{H_{3}}{H_{2}}+\frac{H_{2}H_{4}}{H_{3}H_{6}}\times \frac{\sin\left(\alpha +\psi -90\right)}{\sin\left(90-\alpha \right)}\right)}^{2}{\left(\frac{1}{H_{1}}\right)}^{2}+k_{8}{\left(\frac{H_{2}H_{4}}{H_{3}H_{5}}\times \frac{\cos\left(90-\psi \right)}{\cos\left(\alpha +\beta -90\right)}+\frac{H_{2}H_{4}}{H_{3}H_{6}}\times \frac{\sin\left(\alpha +\psi -90\right)}{\sin\left(90-\alpha \right)}\right)}^{2}{\left(\frac{1}{H_{1}}\right)}^{2}\\ +k_{9}{\left(\frac{H_{2}H_{4}}{H_{3}H_{6}}\times \frac{\sin\left(\alpha +\psi -90\right)}{\sin\left(90-\alpha \right)}\right)}^{2}{\left(\frac{1}{H_{1}}\right)}^{2}+4k_{b1}{\left(\frac{H_{2}H_{4}\cos\left(90-\psi \right)}{H_{3}\cos(\alpha )H_{b1}}\right)}^{2}{\left(\frac{1}{H_{1}}\right)}^{2}+4k_{b2}{\left(\frac{H_{2}H_{4}\cos\left(90-\psi \right)}{H_{3}\cos(\alpha )H_{b2}}\right)}^{2}{\left(\frac{1}{H_{1}}\right)}^{2}\\ \left.+4k_{e1}{\left(\frac{H_{b2}\cos\left(90-\delta \right)}{2H_{e}}\times \frac{H_{2}H_{4}\cos\left(90-\psi \right)}{H_{b2}H_{3}\cos\left(\alpha \right)}\right)}^{2}{\left(\frac{1}{H_{1}}\right)}^{2}+4k_{e2}{\left(\frac{H_{b2}\cos\left(\delta \right)}{2H_{ge}}\times \frac{H_{2}H_{4}\cos\left(90-\psi \right)}{H_{b2}H_{3}\cos\left(\alpha \right)}\right)}^{2}{\left(\frac{1}{H_{1}}\right)}^{2}\right]d_{in}^{2} \end{array}$$

where *F_in_* denotes the input force, and d_in_ symbolizes the input displacement. In addition, k_1_, k_2_, k_3_, k_4_, k_5_, k_6_, k_7_, k_8_, k_9_, k_b1_, k_b2_, k_e1_, and k_e2_ represent the stiffness of compliant springs, including the circular and leaf hinges, as illustrated in Figure 12, Figure 14 and Figure 15.

The input spring stiffness is computed by the following equations:

(33) $$K_{in}=\frac{F_{in}}{d_{in}}$$

(34) $$\begin{array}{l} K_{in}=\left(k_{1}+k_{2}+k_{3}\right){\left(\frac{1}{H_{1}}\right)}^{2}+\left(k_{4}+k_{5}\right){\left(\frac{H_{3}}{H_{2}H_{1}}\right)}^{2}+k_{7}{\left(\frac{H_{2}H_{4}}{H_{1}H_{3}H_{5}}\times \frac{\cos\left(90-\psi \right)}{\cos\left(\alpha +\beta -90\right)}\right)}^{2}\\ +k_{6}{\left(\frac{H_{3}}{H_{2}}+\frac{H_{2}H_{4}}{H_{3}H_{6}}\times \frac{\sin\left(\alpha +\psi -90\right)}{\sin\left(90-\alpha \right)}\right)}^{2}{\left(\frac{1}{H_{1}}\right)}^{2}+k_{8}{\left(\frac{H_{2}H_{4}}{H_{3}H_{5}}\times \frac{\cos\left(90-\psi \right)}{\cos\left(\alpha +\beta -90\right)}+\frac{H_{2}H_{4}}{H_{3}H_{6}}\times \frac{\sin\left(\alpha +\psi -90\right)}{\sin\left(90-\alpha \right)}\right)}^{2}{\left(\frac{1}{H_{1}}\right)}^{2}\\ +k_{9}{\left(\frac{H_{2}H_{4}}{H_{3}H_{6}}\times \frac{\sin\left(\alpha +\psi -90\right)}{\sin\left(90-\alpha \right)}\right)}^{2}{\left(\frac{1}{H_{1}}\right)}^{2}+4k_{b1}{\left(\frac{H_{2}H_{4}\cos\left(90-\psi \right)}{H_{3}\cos(\alpha )H_{b1}}\right)}^{2}{\left(\frac{1}{H_{1}}\right)}^{2}+4k_{b2}{\left(\frac{H_{2}H_{4}\cos\left(90-\psi \right)}{H_{3}\cos(\alpha )H_{b2}}\right)}^{2}{\left(\frac{1}{H_{1}}\right)}^{2}\\ +4k_{e1}{\left(\frac{H_{b2}\cos\left(90-\delta \right)}{2H_{e}}\times \frac{H_{2}H_{4}\cos\left(90-\psi \right)}{H_{b2}H_{3}\cos\left(\alpha \right)}\right)}^{2}{\left(\frac{1}{H_{1}}\right)}^{2}+4k_{e2}{\left(\frac{H_{b2}\cos\left(\delta \right)}{2H_{ge}}\times \frac{H_{2}H_{4}\cos\left(90-\psi \right)}{H_{b2}H_{3}\cos\left(\alpha \right)}\right)}^{2}{\left(\frac{1}{H_{1}}\right)}^{2} \end{array}$$

Later on, E_k_ and E_v_ are integrated into a Lagrange equation.

(35) $$\frac{d}{dt}\left(\frac{\partial L}{\partial {\dot{\varphi }}_{1}}\right)-\frac{\partial L}{\partial \varphi_{1}}=0$$

where E_k_ denotes the kinetic energy, and E_v_ denotes the elastic potential energy.

In which:

(36) $$L\;=\;E_{K}-\;E_{V}$$

Then, in the analytical model, energy is preserved throughout the amplifier’s operation, with modifications translated $\varphi_{1}$ to conduct the computation procedure.

The resolution of the Lagrange equation yields a generalized motion equation.

(37) $$\bar{M}{\ddot{\varphi }}_{i}+\bar{K}\varphi_{i}=0$$

where $\bar{M}$ and $\bar{K}$ denote the equivalent mass and the equivalent stiffness, respectively.

(38) $$\begin{array}{l} \bar{M}=m_{1}{\left(\frac{H_{2}}{2}\right)}^{2}+I_{1}+m_{a}{\left(\frac{H_{2}}{2}\right)}^{2}+m_{H_{3}}{\left(\frac{H_{3}}{2}\times \frac{H_{3}}{H_{2}}\right)}^{2}+I_{H_{3}}{\left(\frac{H_{3}}{H_{2}}\right)}^{2}+m_{H_{4}}{\left(\frac{H_{4}}{2}\times \frac{H_{3}}{H_{2}}\right)}^{2}\\ +I_{H_{4}}{\left(\frac{H_{3}}{H_{2}}\right)}^{2}+m_{H_{5}}{\left(\frac{H_{2}H_{4}}{2H_{3}}\times \frac{\cos\left(90-\psi \right)}{\cos\left(\alpha +\beta -90\right)}\right)}^{2}+I_{H_{5}}{\left(\frac{H_{2}H_{4}}{H_{3}H_{5}}\times \frac{\cos\left(90-\psi \right)}{\cos\left(\alpha +\beta -90\right)}\right)}^{2}\\ +m_{H_{6}}{\left(\frac{H_{2}H_{4}}{2H_{3}}\times \frac{\sin\left(\alpha +\psi -90\right)}{\sin\left(90-\alpha \right)}\right)}^{2}+I_{H_{6}}{\left(\frac{H_{2}H_{4}}{H_{3}H_{6}}\times \frac{\sin\left(\alpha +\psi -90\right)}{\sin\left(90-\alpha \right)}\right)}^{2}\\ +2m_{H_{b1}}{\left(\frac{H_{2}H_{4}\cos\left(90-\psi \right)}{2H_{3}\cos\left(\alpha \right)}\right)}^{2}+2I_{H_{b1}}{\left(\frac{H_{2}H_{4}\cos\left(90-\psi \right)}{H_{b1}H_{3}\cos\left(\alpha \right)}\right)}^{2}+2m_{H_{b2}}{\left(\frac{H_{2}H_{4}\cos\left(90-\psi \right)}{2H_{3}\cos\left(\alpha \right)}\right)}^{2}\\ +2I_{H_{b2}}{\left(\frac{H_{2}H_{4}\cos\left(90-\psi \right)}{H_{b2}H_{3}\cos\left(\alpha \right)}\right)}^{2}+\frac{1}{2}m_{1a}{\left(\frac{H_{b1}}{2}\right)}^{2}{\left(\frac{H_{2}H_{4}\cos\left(90-\psi \right)}{H_{b1}H_{3}\cos\left(\alpha \right)}\right)}^{2}\\ +m_{2a}{\left(\frac{H_{b1}}{2}\right)}^{2}{\left(\frac{H_{2}H_{4}\cos\left(90-\psi \right)}{H_{b1}H_{3}\cos\left(\alpha \right)}\right)}^{2}+4m_{e}{\left(\frac{H_{e}}{2}\times \frac{H_{b2}\cos\left(90-\delta \right)}{2H_{e}}\times \frac{H_{2}H_{4}\cos\left(90-\psi \right)}{H_{b2}H_{3}\cos\left(\alpha \right)}\right)}^{2}\\ +2m_{ge}{\left(\frac{H_{ge}}{2}\times \frac{H_{b2}\cos\left(\delta \right)}{2H_{ge}}\times \frac{H_{2}H_{4}\cos\left(90-\psi \right)}{H_{b2}H_{3}\cos\left(\alpha \right)}\right)}^{2} \end{array}$$

(39) $$\begin{array}{l} \bar{K}=\left(k_{1}+k_{2}+k_{3}\right)+\left(k_{4}+k_{5}\right){\left(\frac{H_{3}}{H_{2}}\right)}^{2}+k_{7}{\left(\frac{H_{2}H_{4}}{H_{3}H_{5}}\times \frac{\cos\left(90-\psi \right)}{\cos\left(\alpha +\beta -90\right)}\right)}^{2}\\ +k_{6}{\left(\frac{H_{3}}{H_{2}}+\frac{H_{2}H_{4}}{H_{3}H_{6}}\times \frac{\sin\left(\alpha +\psi -90\right)}{\sin\left(90-\alpha \right)}\right)}^{2}+k_{8}{\left(\frac{H_{2}H_{4}}{H_{3}H_{5}}\times \frac{\cos\left(90-\psi \right)}{\cos\left(\alpha +\beta -90\right)}+\frac{H_{2}H_{4}}{H_{3}H_{6}}\times \frac{\sin\left(\alpha +\psi -90\right)}{\sin\left(90-\alpha \right)}\right)}^{2}\\ +k_{9}{\left(\frac{H_{2}H_{4}}{H_{3}H_{6}}\times \frac{\sin\left(\alpha +\psi -90\right)}{\sin\left(90-\alpha \right)}\right)}^{2}+4k_{b1}{\left(\frac{H_{2}H_{4}\cos\left(90-\psi \right)}{H_{3}\cos(\alpha )H_{b1}}\right)}^{2}+4k_{b2}{\left(\frac{H_{2}H_{4}\cos\left(90-\psi \right)}{H_{3}\cos(\alpha )H_{b1}}\right)}^{2}\\ +4k_{e1}{\left(\frac{H_{b2}\cos\left(90-\delta \right)}{2H_{e}}\times \frac{H_{2}H_{4}\cos\left(90-\psi \right)}{H_{b2}H_{3}\cos\left(\alpha \right)}\right)}^{2}+4k_{e1}{\left(\frac{H_{b2}\cos\left(\delta \right)}{2H_{ge}}\times \frac{H_{2}H_{4}\cos\left(90-\psi \right)}{H_{b2}H_{3}\cos\left(\alpha \right)}\right)}^{2} \end{array}$$

The natural frequency is identified by the following equation:

(40) $$f=\frac{1}{2\pi }{\left(\frac{\bar{K}}{\bar{M}}\right)}^{0.5}$$

### 3.3. Analytical Results Verification

**Figure 16:**
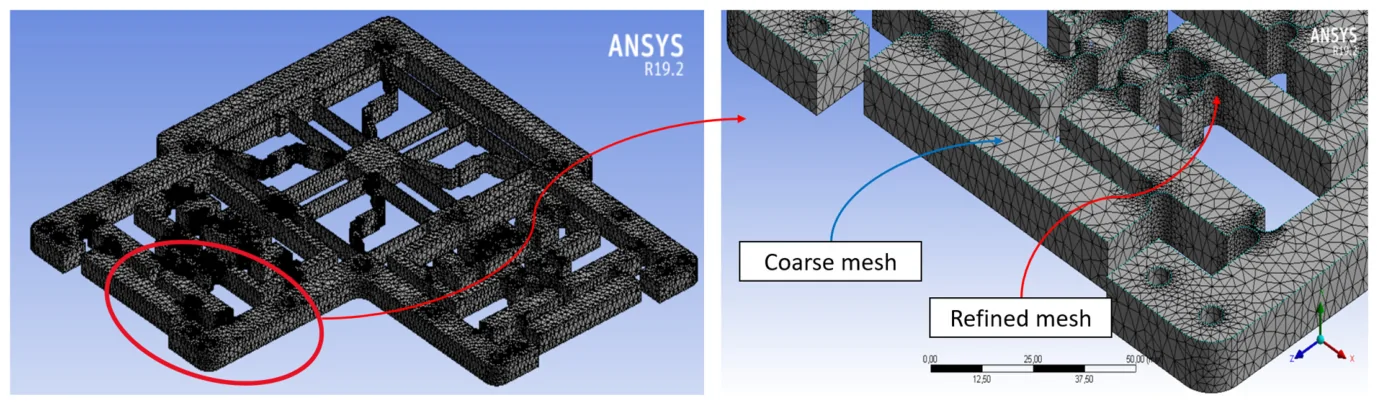
Mesh dividing structure of the proposed XY mechanism.

**Figure 17:**
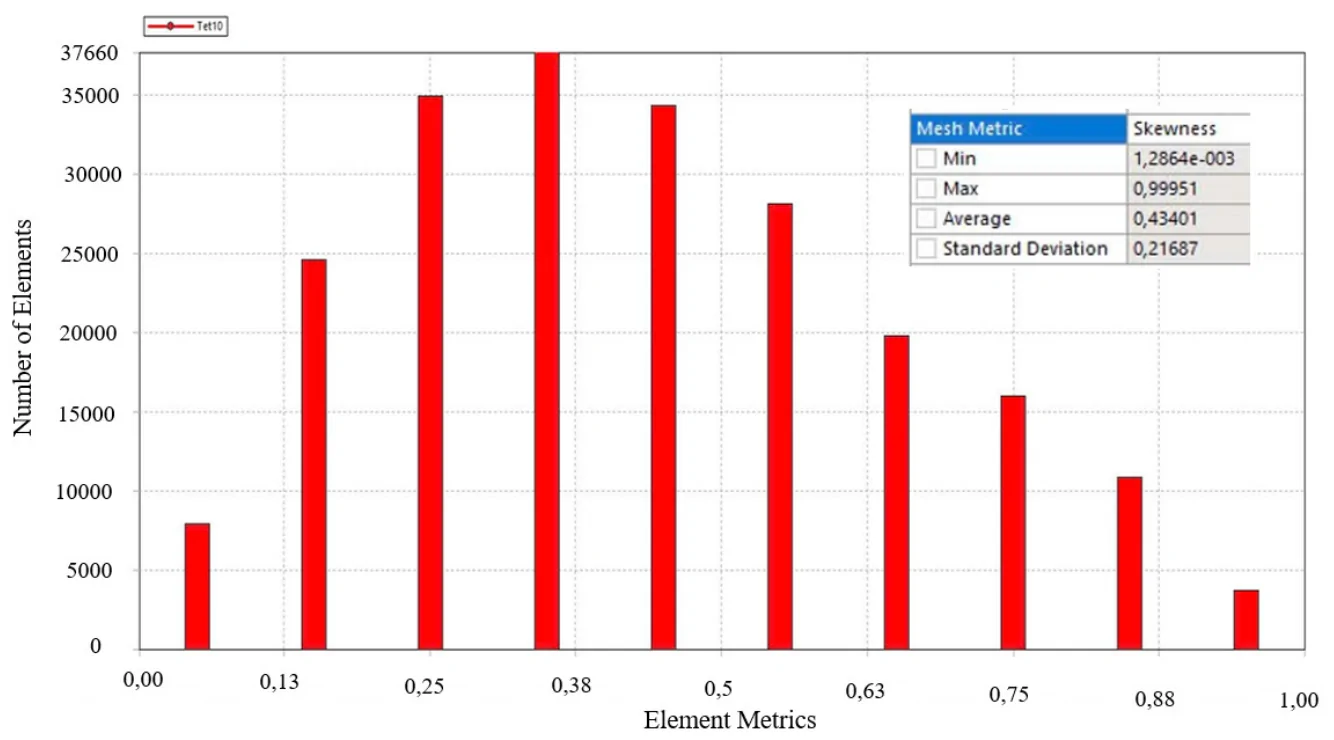
Skewness standard chart of the suggested positioner.

**Figure 18:**
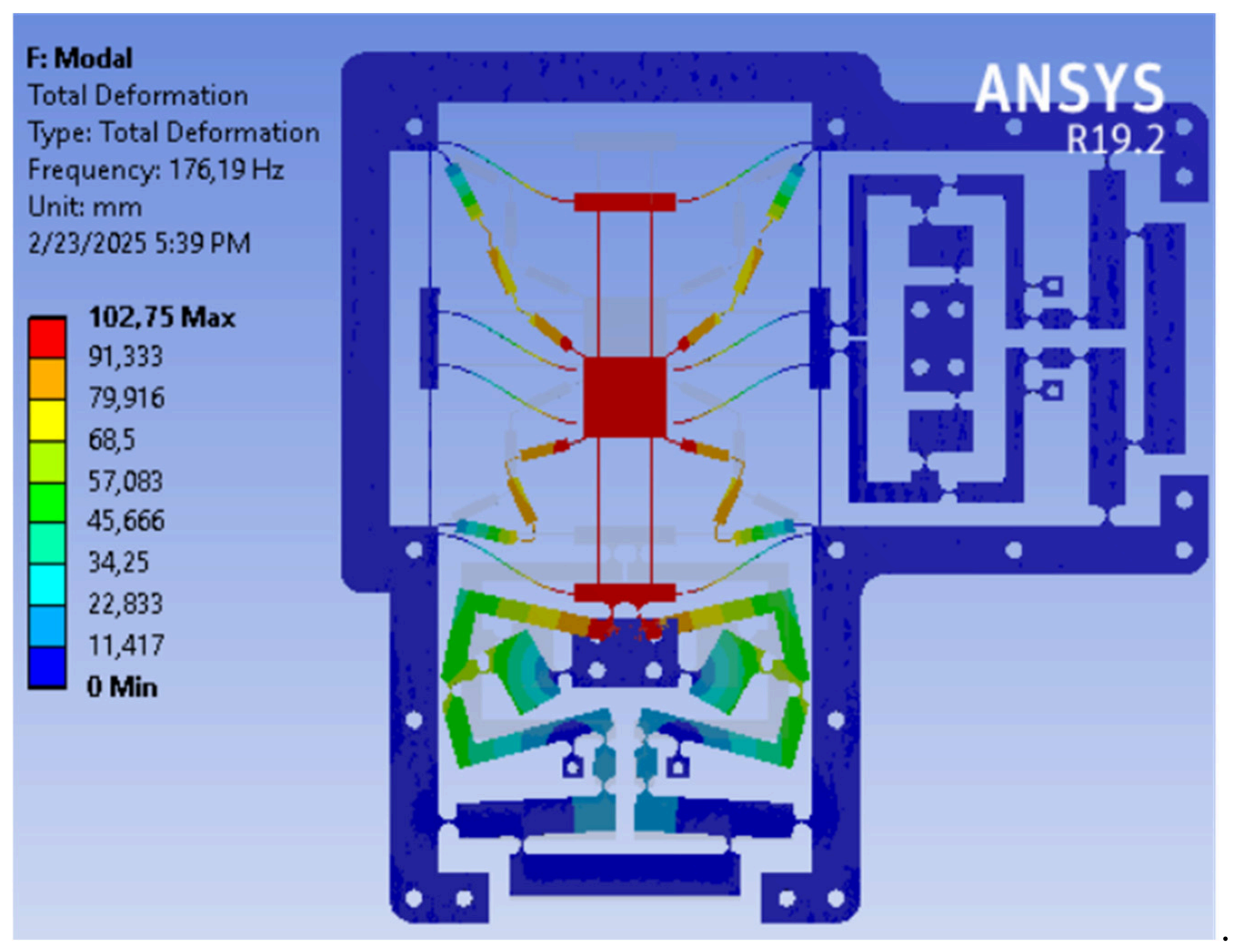
The primary resonant frequency of the primary structure.

The resonant frequency, according to the proposed analytical method, was validated by FEA. Specifically, the analytical frequency result based on Equation (40) was 180.14 Hz. Firstly, to validate the analytical result, a 3D design model was built using a coarse mesh. Secondly, the flexure hinges were refined in order to improve the calculation accuracy, as demonstrated in Figure 16. The Skewness criterion was utilized to evaluate mesh quality, revealing a moderate value of 0.43401, as illustrated in Figure 17. The result shows that the average value obtained indicates a very good mesh quality based on the Skewness standard [38]. The FEA frequency result is illustrated in Figure 18.

The discrepancy between the analytical and FEM approaches was 2.24%, as demonstrated in Table 3. The inaccuracy indicates that the suggested analytical approach is both advantageous and dependable for rapidly evaluating the initial output characteristics of the suggested platform.

In addition, the absolute ratio of the unexpected displacement to the desired displacement was used to define the parasitic motion error [39]. Based on the FEA results, the developed positioner’s parasitic motion error was around 0.28% (less than 1%), as shown in Table 4. Therefore, the parasitic motion error has minimal effect on output displacement accuracy.

### 3.4. Working Stroke Analysis

The operational stroke of the proposed XY positioner can be determined by multiplying AR_1_ and AR_2_. This estimate omits the effects of axial stress and shearing and focuses solely on bending tension. Therefore, the maximum stress (σ_t_) is constrained by using Equation (41).

(41) $$Max\left(\sigma_{t}\right)\leq \frac{\sigma_{y}}{S_{c}}$$

where *σ_y_* signifies the material’s strength, whereas the safety coefficient is represented by *S*_c_. In addition, *S*_c_ is proposed to be selected at 1.5 to ensure the material’s strength of the developed positioner.

The maximal stress occurs when the maximal angle displacement $\varphi_{\max}$ attains its peak value.

Consequently, the maximal stress computation is conducted by the subsequent formulation.

(42) $$\sigma_{r}^{\max}=\frac{4Er^{2}K_{c}}{f\left(\gamma \right)t^{2}}\times \varphi_{\max}$$

where *K*_c_, $f\left(\gamma \right)$, and γ are the dimensionless focus parameter, flexure parameter, and dimensionless geometry parameter, respectively. The equations for calculating *K*_c_, $f\left(\gamma \right)$ and γ are expressed as follows:

(43) $$K_{c}={\left(1+\gamma \right)}^{\frac{9}{20}}$$

(44) $$\gamma =\frac{t}{2r}$$

(45) $$f\left(\gamma \right)=\frac{1}{2\gamma +\gamma^{2}}\times \left(\frac{3+4\gamma +2\gamma^{2}}{\left(1+\gamma )(2\gamma +\gamma^{2}\right)}+\frac{6(1+\gamma )}{2\gamma +\gamma^{2}}\times \tan^{-1}\sqrt{\frac{2+\gamma }{\gamma }}\right)$$

The maximal deformation angle ($\varphi_{\max}$) is calculated based on the $d_{in}^{\max}$ (maximum input displacement) and the output displacement of the movement platform ($AR\times d_{in}^{\max}$). More specifically, the formula for determining ($\varphi_{\max}$) is expressed by the following equation:

(46) $$\varphi_{\max}=\frac{AR\times d_{in}^{\max}}{H_{6}}$$

Then, $d_{in}^{\max}$ can be ascertained by replacing Equations (43) and (44) into Equation (42), while simultaneously accounting for Equations (45) and (46).

(47) $$\frac{\sigma_{y}}{S_{c}}\geq \frac{4Er^{2}K_{c}}{f\left(\gamma \right)t^{2}}\times \frac{AR\times d_{in}^{\max}}{H_{6}}$$

(48) $$d_{in}^{\max}\leq \frac{\sigma_{y}f\left(\gamma \right)t^{2}H_{6}}{4S_{f}Er^{2}K_{c}AR}$$

The maximal displacement was calculated by replacing the values of the mechanical parameters of the AL 7075-T6 material into Equation (48), and the $d_{in}^{\max}$ value was calculated to be 23.78 μm. Therefore, the maximum XY working stroke of the mechanism is 346.75 × 346.75 μm. Based on Equation (48), the working travel of the developed XY positioner is roughly 346 µm × 346 µm.

### 3.5. Factor Optimization of a Rana Macrodactyla-Motivated XY Positioner

#### 3.5.1. Optimal Problem Formulation

The proposed XY positioner works according to the elastic deformation of compliant hinges. Therefore, the thicknesses of compliant joints and the lengths between flexure hinges are the main design variables affecting the structural responses of the XY platform. A precise locating system for a nanoindentation testing instrument benefits from a higher frequency, which enhances responsiveness and minimizes the risk of motor resonance. Consequently, the resonant frequency is an important attribute of the offered structure. As illustrated in Figure 6 and Table 1, the important parameters, *U* and *L*, are the thicknesses of the right-circular joints at the lever amplification mechanism and the double-rocker amplification mechanism, respectively. In addition, *Q* is the thickness of the compliant leaf joints at an equivalent leading structure, and P is a Rana macrodactyla-inspired leaf hinge thickness. G and S are the distances between two right-circular hinges at the lever and double-rocker amplification mechanisms, respectively. In addition, rather than a universal requirement for nanoindentation devices, the lower-bound constraint of 195 Hz was chosen as an engineering design threshold for optimization. The analytical and FEA models indicated initial first resonance frequencies of 180.14 Hz and 176.19 Hz, respectively. Therefore, 195 Hz was chosen to maintain a workable solution space within the given geometrical constraints while requiring a significant improvement in dynamic response compared with the initial design. Optimization was executed within the geometry that had already been selected so as to provide considerable displacement amplification, while the natural frequency was subsequently enhanced in compliance with the designated geometric design space and a minimum frequency requirement. As a result, the optimal design is illustrated in the list below.

Seek the key variable vector: *X* = [*U*, *L*, *Q*, *P*, *G*, *S*].

(49) Maximize the resonant frequency: f(X)

(50) Constraint: f(X)≥195 (Hz)

Limitations of the main design variables:

(51) 25≤G≤3012≤S≤140.65≤U≤0.750.5≤L≤0.650.5≤Q≤0.650.5≤P≤0.65

**Figure 19:**
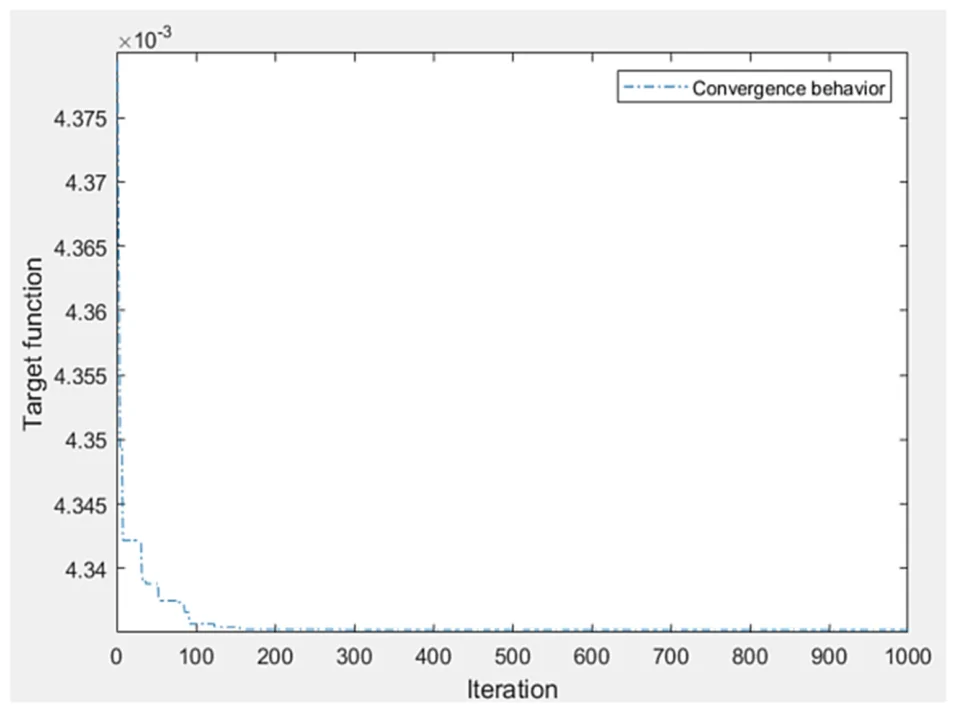
Convergence plot of the Firefly algorithm.

More specifically, the analytical modelling for calculating the resonant frequency was established in Equation (40). The Firefly algorithm was coded to define the optimal parameters based on MATLAB R2021b. The convergence plot of the Firefly algorithm based on a chain of analytical equations is depicted in Figure 19. The achieved result shows that the optimal frequency according to the analytical approach was 197.1 Hz. The optimization results of the main design variables are found at *G* = 25 mm, *S* = 14 mm, *U* = 0.75 mm, *L* = 0.65 mm, *Q* = 0.5 mm, and *P* = 0.6 mm.

#### 3.5.2. Validation of Optimal Results by FEA

**Figure 20:**
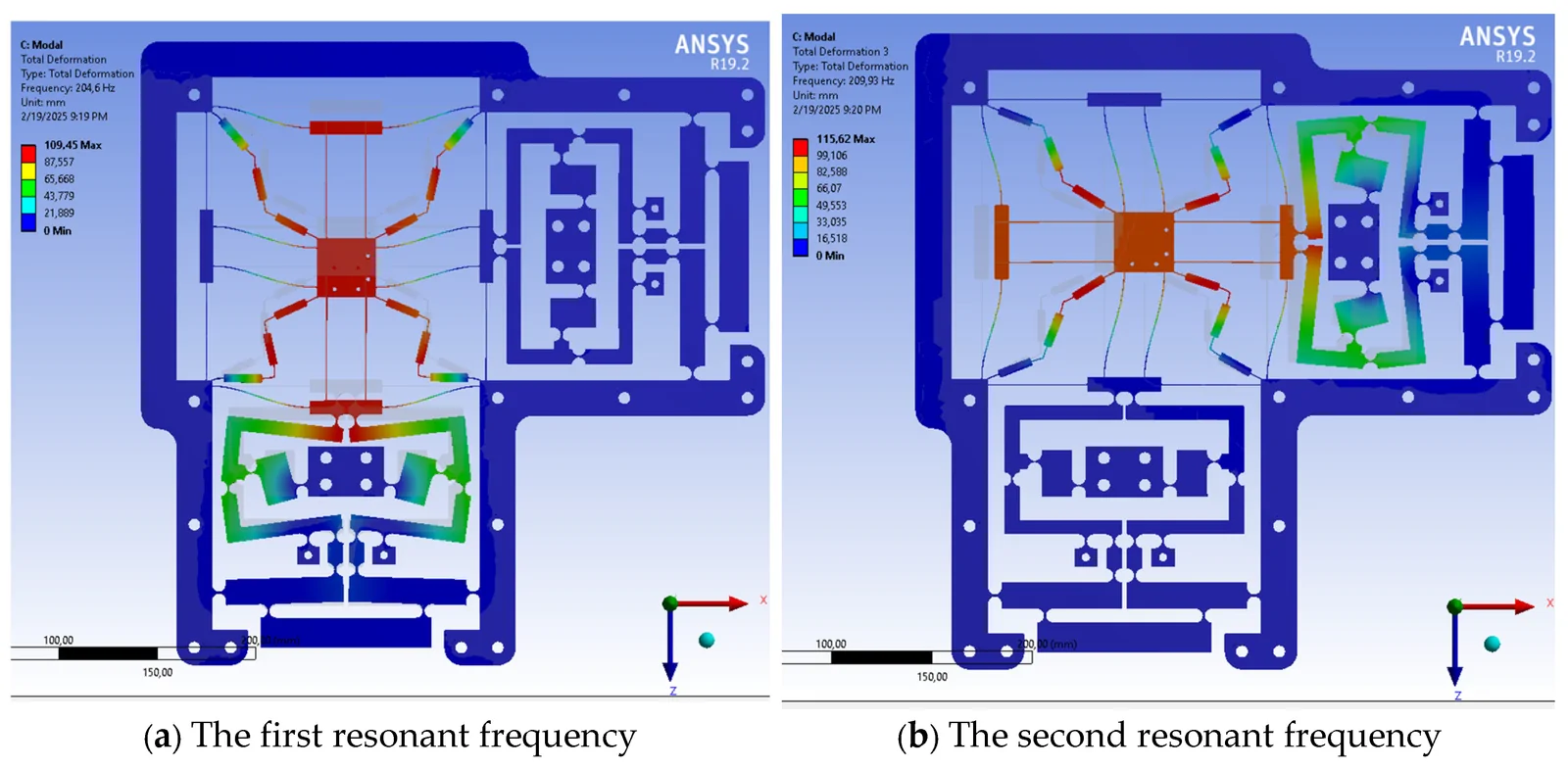

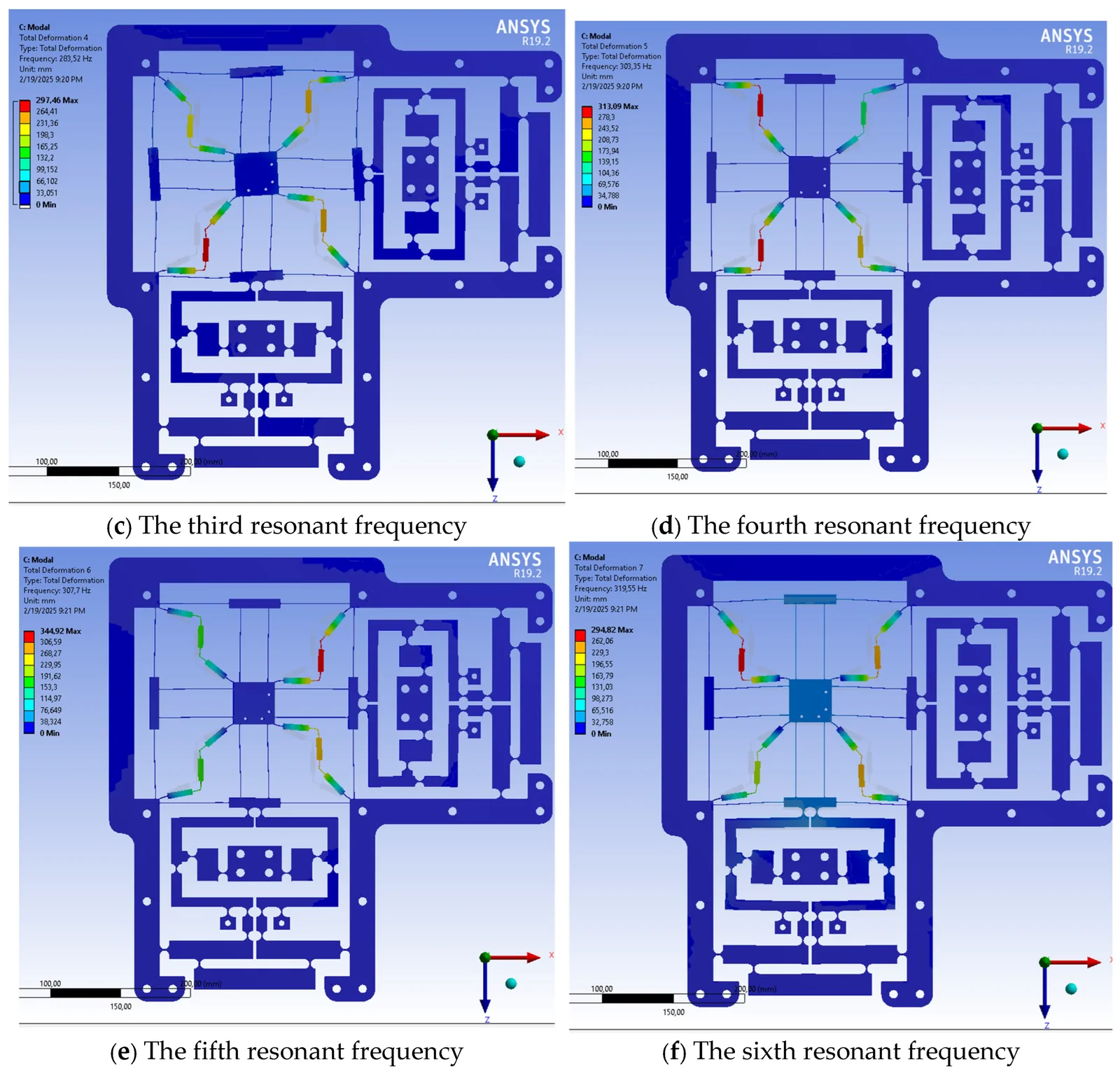
The values of the six optimization frequencies according to the FEM method.

An optimized 3D design was created to validate the results derived from the Firefly algorithm’s optimization factors. The primary resonant frequency’s best result, as indicated by FEA, was 204.6 Hz, illustrated in Figure 20a. The result shows that the achieved frequency of the proposed positioner after the optimal procedure is a suitable frequency and satisfies the preliminary conditions. As a result, the error between the FEA and optimization results was 3.8%, as demonstrated in Table 5. The accuracy showed that the hybrid approach was effective and reliable. In addition, based on the optimal parameters, the natural frequency results for the first six modes, according to FEA, were 204.6 Hz, 209.93 Hz, 283.52 Hz, 303.35 Hz, 307.7 Hz, and 319.55 Hz, correspondingly, as depicted in Figure 20a–f.

## 4. Experimental Research

### 4.1. Prototype Manufacturing and Experimental Layout

To assess and confirm the reliability of the analytical models and the effective optimization approach, the optimal XY positioner was fabricated by CNC wire-discharged machining based on the obtained optimization results of the main design variables.

**Figure 21:**
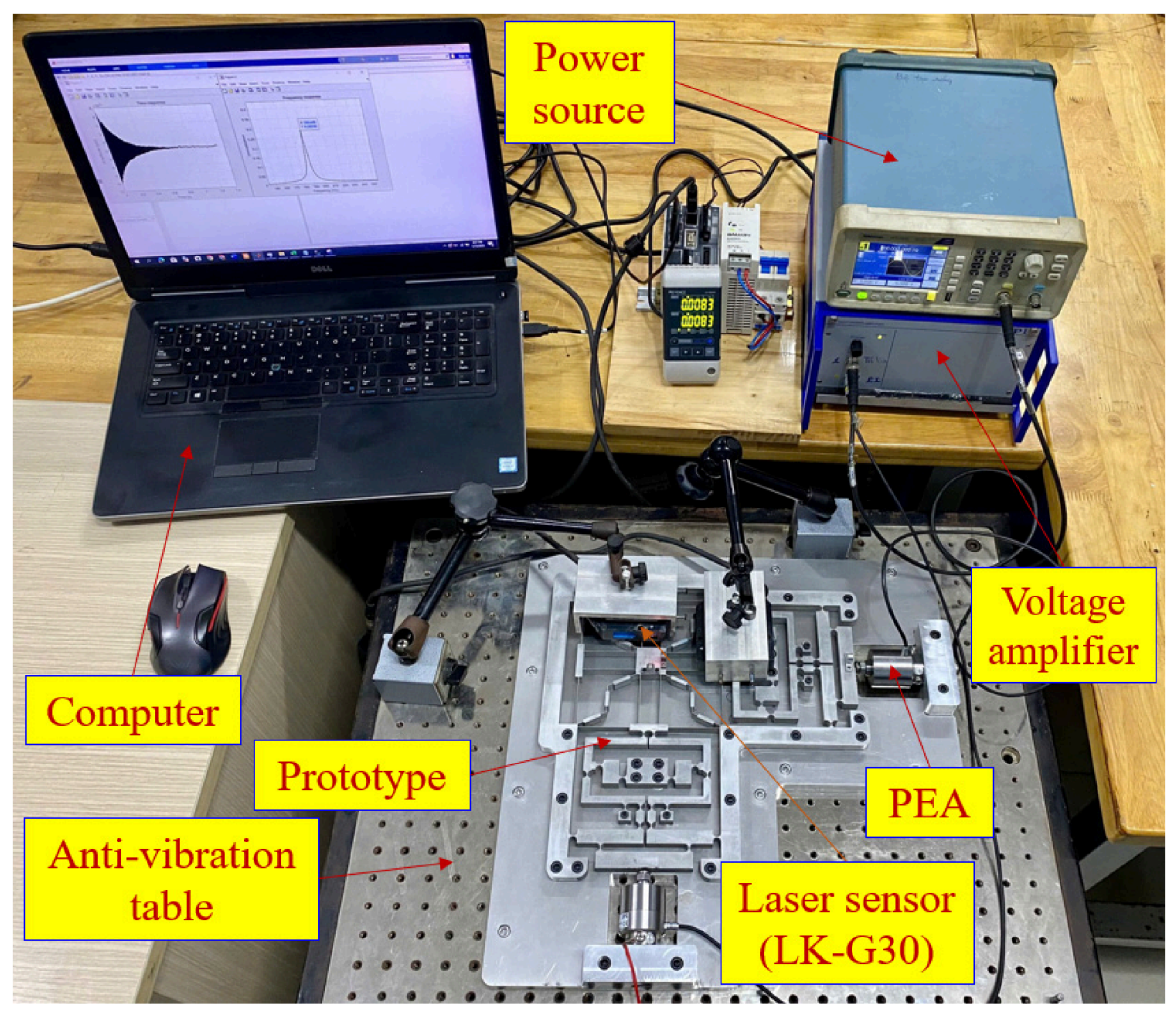
Experimental setup layout for testing the output attributes of the XY mechanism.

The experimental layout for measuring the first resonant frequency of the proposed prototype, based on the hammer test approach, is demonstrated in Figure 21. More specifically, the fabricated XY model and the testing instruments were installed on an anti-vibration table to ensure measurement accuracy. The following devices were used to support the measurement process. Initially, two PZT actuators (PI.225.10, Germany) were used, with a maximum displacement of 15 µm and a maximum force of 12,500 N. Secondly, with a maximum measuring dimension of 30 mm and an accuracy of 0.05 µm, the second instrument comprised two displacement laser sensors, namely, the LK-G30 (Keyence, Japan). Furthermore, the laser sensor accurately measured vibrations of the central stage in the X and Y axes with an accuracy of 0.05 µm, a reference range of 30 mm, and a measuring range of ± 5 mm. The third instrument, with a power rating of 550 W, was the PZT Controller (Voltage Amplifier, PI, Germany) for controlling the PZT actuator. The final instrument was a power source (Tektronix AFG1022) that provides the frequency and voltage for controlling the PZT actuator. In addition, the experiment process was conducted in the following steps. The PZT actuator exerted displacement on the prototype. Subsequently, two laser displacement sensors captured output signals for gathering data based on laser-controlling software. To determine the primary resonant frequency, the gathered data were analyzed using the rapid Fourier transform algorithm, executed in MATLAB R2021b.

The current study mainly aimed to assess the input–output displacement characteristics rather than perform a specific positioning repeatability test, even though the displacement measurements were repeated five times for each motion axis. Therefore, a quantitative positioning repeatability value should not be deduced without a systematic repeated target-position test. However, the consistency of the repeated measurements provides early data on the reproducibility of the displacement response. Furthermore, the LK-G30 laser displacement sensors’ 0.05 µm accuracy characterizes the measurement system’s performance rather than the positioning resolution of the suggested positioner. The PZT actuator’s minimum controllable displacement, driving electronics, measurement noise, PZT hysteresis and creep, as well as the control method, all affect the system’s achievable positioning resolution. This study does not quantitatively report positional resolution because a dedicated minimum-increment-motion experiment was outside the scope of the current validation. To measure repeatability and positioning resolution more precisely, future studies will include minimum-increment-motion experiments under closed-loop PZT actuation and repeated point-to-point positioning tests.

### 4.2. Experimental Results and Discussion

**Figure 22:**
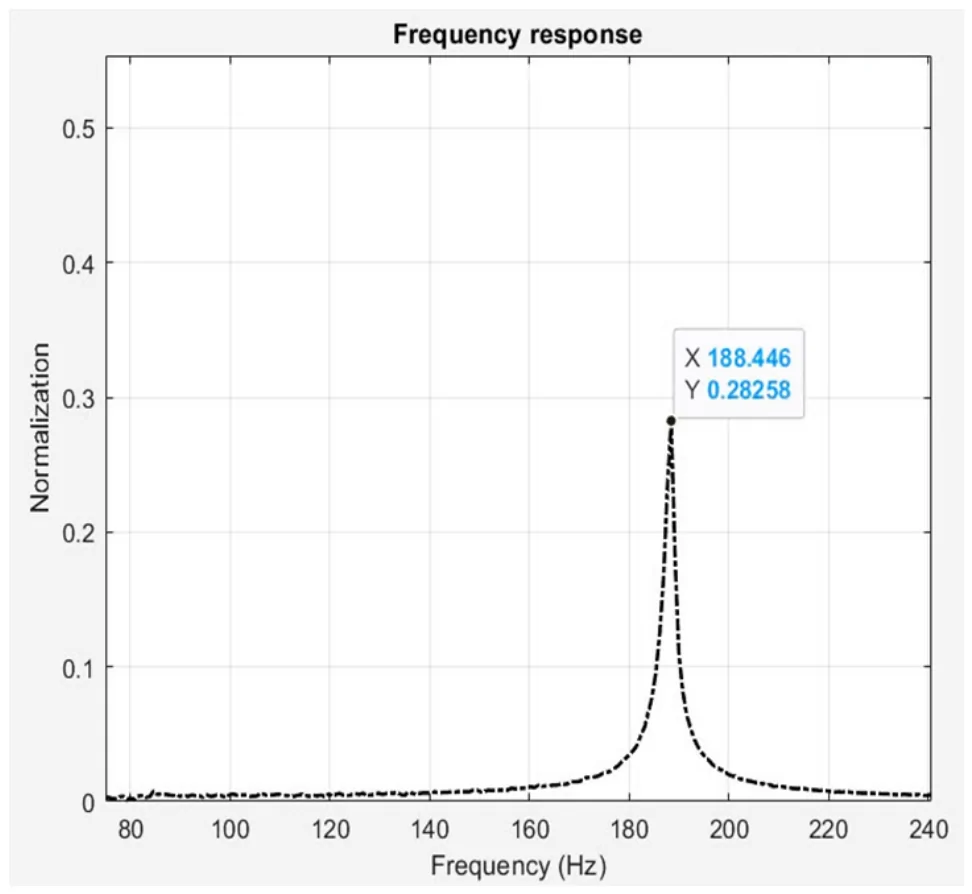
The experiment’s first natural frequency.

To verify the proposed prototype’s initial resonant frequency, the positioner’s optimal FEA frequency, determined by Ansys software R19.2, was 204.6 Hz. The experiment’s first natural frequency outcome was 188.446 Hz, as portrayed in Figure 22. According to the achieved FEA and experimental results, the inaccuracy between the two approaches was 8.6%, as demonstrated in Table 6. The experimental error indicates that the resonant frequency of the structure was comparable to that of the FEM results. Therefore, the results are reasonable and reliable.

**Figure 23:**
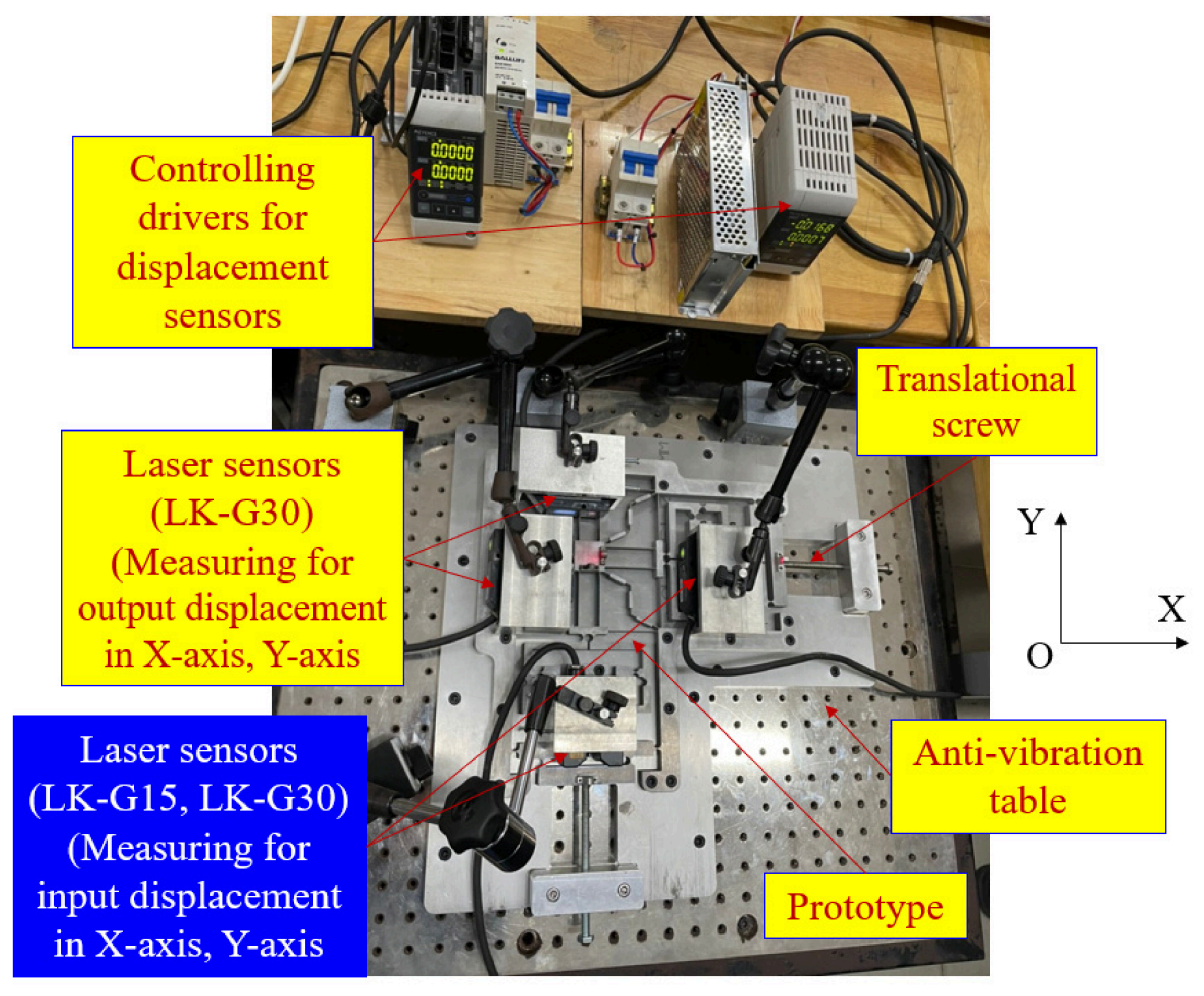
Device layout to measure the output displacement of the developed platform.

**Figure 24:**
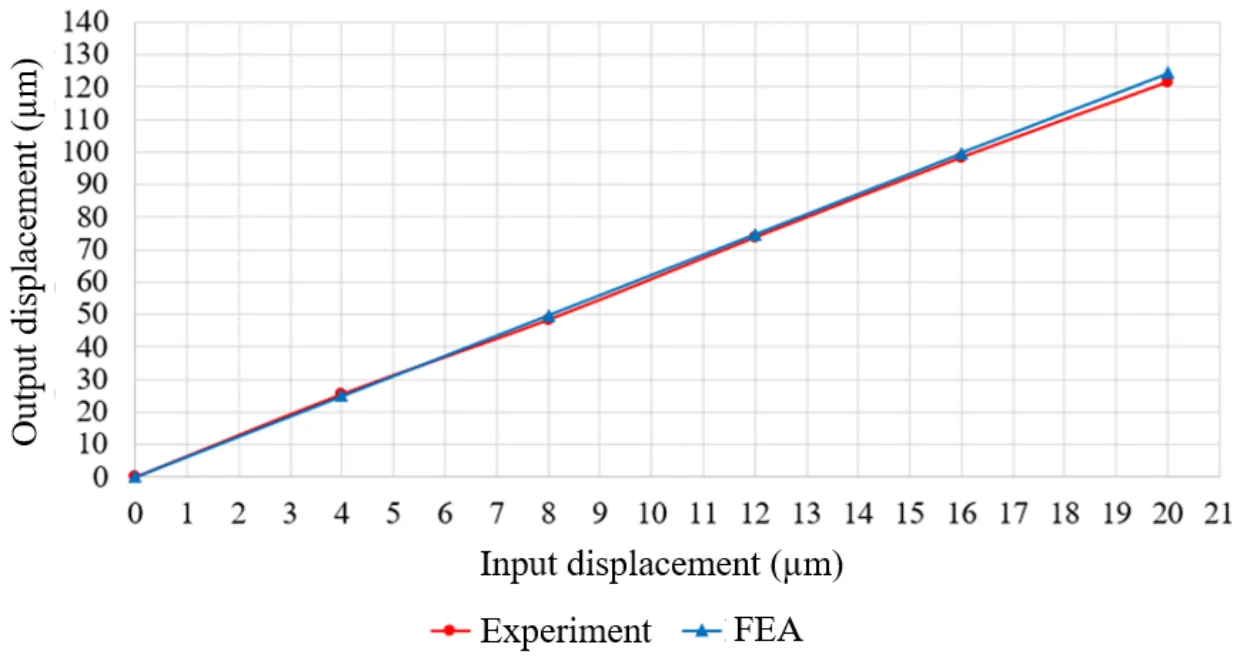
Relationship of input–output deformations in the X-axis.

**Figure 25:**
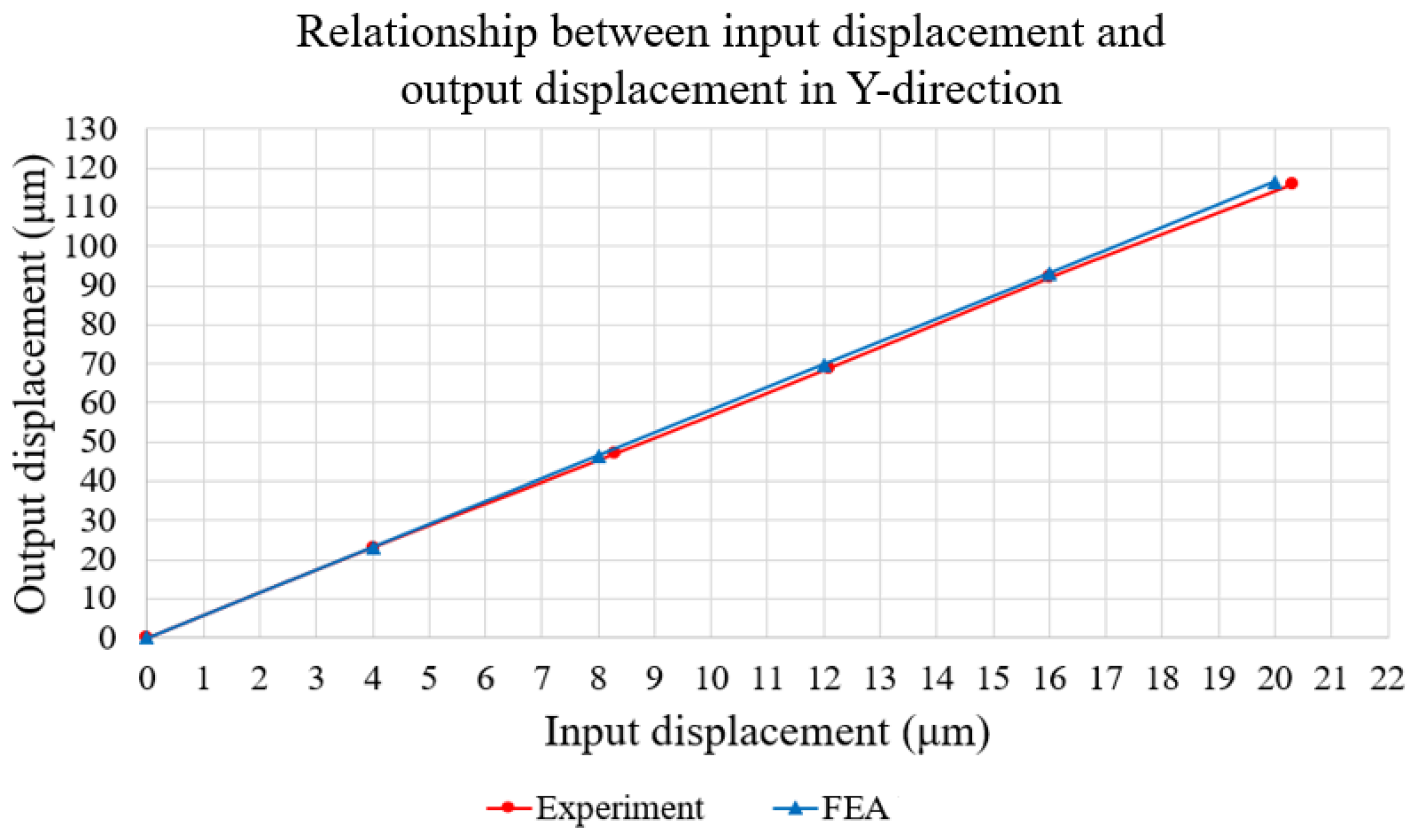
Relationship of input–output deformations in the Y-axis.

In addition, due to the limited range of the PEA’s displacement, measurements of the constructed positioner’s input–output displacement values were carried out using translational screws and displacement sensors. As shown in Figure 23, the measurement system was experimentally configured to evaluate the developed positioner. Displacement data in both the X and Y axes were collected through five repeated measurements for each axis. Using both experimental results and FEA simulations, linear correlations between the input–output displacement values were derived, as presented in Figure 24 and Figure 25. These correlations confirm the positioner’s ability to maintain high linearity across both motion axes. Table 7 summarizes a comparative analysis between the developed design and selected prior studies. More specifically, as Table 7 indicates, the recommended positioner should not be construed as enhancing each distinct performance parameter. Instead, it reflects a balanced design across displacement augmentation, effective stroke, and natural frequency. This behaviour aligns with the inherent stiffness-stroke compromise of compliant positioning systems: increasing flexural compliance and displacement amplification typically increases achievable travel while reducing structural stiffness, thereby lowering the natural frequency. Consequently, the current design emphasizes a substantial amplification ratio and operational stroke while maintaining a resonance frequency appropriate for the specified precision-positioning application.

Based on the results, the mechanism developed in this study demonstrates a balance among a high displacement amplification ratio, a suitable frequency, and linear motion.

## 5. Conclusions

A new bioinspired flexure stage with a new amplification mechanism was developed and validated. Specifically, this study develops a novel positioner according to a new amplifier mechanism of a one-lever structure and a double-rocker structure, with the flexural movement characteristics of the Rana macrodactyla leg profile integrated into a central mobile platform.

This study aims to achieve a compact design, a suitable initial natural frequency, satisfactory linear motion, and a significant displacement amplification ratio. Therefore, a hybrid approach of static-dynamic analysis, the Lagrange technique, and the FEM method was employed to evaluate and validate the output attributes of the proposed positioner. The achieved results indicate that the difference between the analytical method and FEM was 2.24%. The observed error confirms that the proposed analytical procedure is both reliable and efficient for the rapid evaluation of the initial output attribute of the suggested platform. The operating travel of the proposed design was approximately 346 µm × 346 µm. In addition, the positioner developed in this study achieved an analytical amplification ratio of 14.58.

Thirdly, to enhance the output attribute, the Firefly optimization technique was employed to optimize the significant factors of the proposed positioner. The optimized components were utilized to build a 3D design of the positioner to validate the optimal outcome based on FEA. The optimization and FEA results were 197.1 Hz and 204.6 Hz, respectively. In addition, the difference between 02 approaches was 3.8%.

Fourthly, the optimized prototype was fabricated in accordance with the optimal parameters of the design variables, while the experimental procedure validated the optimization and FEA results. The advanced mechanism’s experimental frequency was 188.4 Hz. The difference between FEA and the experimental outcomes was 8.6%. In addition, the relationships among the input–output displacement values in the X- and Y-axes were investigated. The strong experimental linear correlation suggests that the developed structure can serve as a reliable configuration for precise transmission mechanisms and a nanoindentation-checking locating system for assessing mechanical characteristics. Finally, the main results of the present research were compared with several other studies.

The developed positioner demonstrates a strong potential for nanoindentation applications and other precision positioning systems. Future research would involve developing a real-time checking instrument that incorporates a proposed positioner to observe and measure the mechanical characteristics of the bio-specimen. In addition, new compact compliant XY positioners with good response would be considered for development and integration with advanced sensors and control systems for precise positioning.

## Figures and Tables

**Table 1:** Geometrical factors of the proposed structure (unit: mm).

Sign	Worth	Sign	Worth
j	30	a	317
k	24	b	317
m	7	*L*	0.5 ≤ *L* ≤ 0.65
n	39	c	136
i	30	d	136
*U*	0.65 ≤U ≤ 0.75	z	37
*G*	25 ≤ *G* ≤ 30	*Q*	0.5 ≤ *Q* ≤ 0.65
h	64	*P*	0.5 ≤ *P* ≤ 0.65
e	18	*S*	12 ≤ *S* ≤14

**Table 2:** The initial dimension factors for the PRBM scheme.

Sign	Worth	Unit	Sign	Worth	Unit
H1	25	mm	R	3.5	mm
H2	68.15	mm	t_c_	0.75	mm
H3	14	mm	t_rk_	0.65	mm
H4	48	mm	α	52	°
H5	25	mm	β	62	°
H6	65	mm	ψ	78	°
H_b1_	49.5	mm	h	12	mm
H_b2_	52.88	mm	b	0.5	mm

**Table 3:** Confirmation of the analytical vs. FEM results.

Characteristic	Analytical Result	FEM Result	Discrepancy (%)
*f*(Hz)	180.14	176.19	2.24

**Table 4:** Results of input displacement, output displacement, and parasitic motion error.

No.	Input Displacement	Output Displacement (Y)	Output Displacement (X)	Parasitic Motion Error (%)
1	2	17.038	4.77 × 10^−2^	0.27996
2	4	34.075	9.5401 × 10^−2^	0.27997
3	6	51.113	0.1431	0.27997
4	8	68.15	0.1908	0.27997
5	10	85.188	0.2385	0.27997
6	12	102.23	0.2862	0.27996
7	14	119.26	0.3339	0.27998
8	16	136.3	0.3816	0.27997
9	18	153.34	0.4293	0.27997
10	20	170.38	0.47701	0.27997
11	22	187.41	0.52471	0.27998
12	24	204.45	0.5724	0.27997
13	26	221.49	0.62011	0.27997
14	28	238.53	0.6678	0.27996
15	30	255.56	0.7155	0.27997
16	32	272.6	0.76321	0.27997
17	34	289.64	0.81091	0.27997
18	36	306.68	0.8586	0.27997
19	38	323.71	0.9063	0.27997
20	40	340.75	0.95401	0.27997
21	42	357.79	1.0017	0.27997
22	44	374.83	1.0494	0.27997
23	46	391.87	1.0971	0.27997
24	48	408.9	1.1448	0.27997
25	50	425.94	1.1925	0.27997

**Table 5:** Comparison of FEA and optimal results.

	Optimization	FEA	Error
*f* (Hz)	197.10	204.6	3.8%

**Table 6:** Difference between experimental and FEA results.

Response	Experiment	FEA	Error
f (Hz)	188.4	204.6	8.6%

**Table 7:** Comparison of the present study with previous studies.

Research	Frequency (Hz)	Amplification Ratio	Working Stroke
Li et al. [40]	66.79	5.54	110 µm × 110 µm
Wu et al. [29]	348.31	7.78	169.6 µm × 165.3 µm
Zhang et al. [41]	222	4.7	127 µm × 127 µm
Wang et al. [42]	253.6	5.46	55.4 µm × 53.2 µm
The proposed design	204.6	14.58	346 µm × 346 µm

## Data Availability

The original contributions presented in the study are included in the article; further inquiries can be directed to the corresponding author.
